# Automated Determination of the Molecular Substructure from Nuclear Magnetic Resonance Spectra Using Neural Networks

**DOI:** 10.1021/acs.jcim.5c00499

**Published:** 2025-08-13

**Authors:** Shiyun Liu, Jacqueline M. Cole

**Affiliations:** 1 Cavendish Laboratory, Department of Physics, 2152 University of Cambridge, J. J. Thomson Avenue, Cambridge CB3 0HE. U.K.; 2 Science and Technology Facilities Council, Harwell Science and Innovation Campus, Didcot, Oxfordshire OX14 0FA, U.K.

## Abstract

Nuclear magnetic resonance (NMR) spectroscopy is an indispensable tool for determining the structural characteristics of a molecule by analyzing its chemical shifts. A wealth of NMR spectra therefore exists and continues to amass on a daily basis, at an ever-increasing rate owing to the progressive automation of chemical analysis. This growth and automation have led to the data analysis step in NMR spectroscopy becoming the main bottleneck in the structural characterization of a new chemical compound. In particular, the data interpretation step is slow and prone to error as it requires manual examination by a suitably trained scientist. Machine learning (ML) methods could overcome this bottleneck, pending that they can automatically correlate the collection of peaks in an NMR spectrum with the substructure of its subject molecule. This study explores the art of the possible using three types of ML methods that are based on neural-network architectures: a multilayer perceptron (MLP) + long short-term memory (LSTM) neural network, a convolutional neural network (CNN), and an MLP + recurrent neural network (RNN). NMR spectrum–structure correlations were encoded into each type of neural network using two forms of molecular representation, one employing functional groups and the other using a novel neighbor-based method. These models were trained on 34,503 and 17,311 experimental ^13^C and ^1^H NMR spectra, respectively. The influence of incorporating metadata about experimental conditions (NMR field strength, temperature, and solvent) into the neural-network model was also investigated. The models incorporated coupling constants as a proxy for spectral intensities in the case of ^13^C NMR spectra. We found that the MLP + LSTM model achieved the highest accuracy (88%) when trained on ^13^C NMR spectra and incorporating experimental metadata (compared to 77% without incorporating it). While the CNN model performance was slightly lower (86% accuracy), it determined molecular substructures in one-third of the computational run time compared to the MLP + LSTM model. Thus, the CNN model emerged as the practically best model when considering performance, time, and cost.

## Graphical abstract

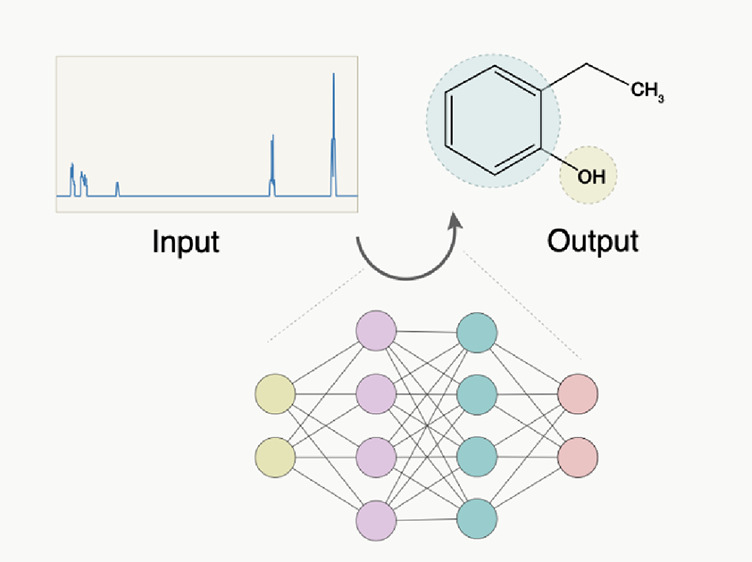

## Introduction

Spectroscopy is one of the most widely used analytical techniques for investigating the atomic and molecular structure of chemical compounds. Spectral analysis is crucial for determining the structure of a compound. It forms the basis for countless practical applications such as drug discovery, food safety, and metabolomics as well as fundamental research topics, e.g., structural chemistry, material characterizations, and protein structure.^1^ The determination of spectrum–structure correlations lies at the heart of this analysis, whereby spectral features (peak profiles and shifts) need to be mapped to underlying structural information. The decoding of the spectrum usually requires scientific expertise to match structure from vast databases, a method that is slow and prone to bias and error. Given the increasing pace of spectroscopic data acquisition, owing to technological advances in experimental automation, there is an urgent need to overcome the time-consuming and error-prone limitations of manual data analysis of spectra.

One promising opportunity to overcome these limitations concerns the latest developments in machine learning (ML).^2–4^ ML methods can be used to automate spectral data analysis as well as enhance the accuracy and efficiency of the whole process. For example, the power of using ML algorithms to realize key milestones toward automated structure determination has already been demonstrated for several forms of materials characterization: mass spectroscopy,^3,5–7^ IR spectroscopy,^2,8–10^ and NMR spectroscopy.^11–13^ Recently, a review by Lu et al. summarized the use of deep learning-based methods in deciphering spectrum–structure correlations and highlighted research challenges.^4^

Last year, Kuhn et al. reviewed the current applications of ML methods to the data analysis of NMR spectra.^14^ NMR spectroscopy is the subject of this work since ^1^H and ^13^C NMR spectra are a key source of chemical analysis for most organic molecules; yet, NMR data analysis suffers from the fundamental issue of needing to process a large volume of spectra, despite the use of computer-assisted structure elucidation (CASE) methods.^15,16^ While CASE programs help to automate and enhance the efficiency of spectral analysis, they tend to employ rule-based expert systems, with limited consideration being given to ML algorithms.

In 2020, Martínez-Treviño et al. published a study that compared various ML algorithms for structural elucidation of natural product (NP) classes; using similarities in structures as a basis, they explored the possibility of class prediction of eight NPs via 1D ^13^C NMR data.^17^ A year later, Specht et al. presented a type of binary classification algorithm, using the support vector clustering (SVC) method, to introduce new automated methods that reveal the structural groups in unknown compounds (pure or mixtures) based on their ^1^H and ^13^C NMR spectra.^12^ This study employed around 1000 pure compounds that contained carbon (C), oxygen (O), and hydrogen (H) with an upper bound on molar mass (160 g mol^–1^) and a limit on number of carbon atoms (eight) per molecule. This preliminary work confirmed the feasibility of using ML methods to uncover the chemical substructure from 1D NMR spectra without manual implementation. Subsequently, Li et al. explored optimal ML methods for identifying substructures in organic compounds.^18^ They compared the prediction capabilities of traditional ML algorithms like support vector machines (SVM) and *k*-nearest neighbors (KNN) with deep learning models like recurrent neural networks (RNNs). SVM algorithms attempt to find the best decision boundary to separate different classes of data with maximum margin whereas KNN is a clustering algorithm.^3^ KNN classifies data by looking at the data points on the majority class of their *k*-nearest neighbors. In contrast, RNNs are a class of artificial neural networks (ANN) designed to handle sequential data by using its previous inputs as inputs for the next steps. The RNN model exhibited the best performance across all evaluation metrics. However, this study was limited by the relatively small size of the training data employed.

While this was important work, 1D NMR spectra contain overlapping peaks and complex splitting patterns, which makes it harder to decipher the spectral data. In contrast, 2D NMR spectra contain more detailed correlations and provide better spectral resolution. Therefore, researchers have applied convolutional neural networks (CNNs) to 2D NMR spectra instead of 1D spectra for identifying molecular substructures^19^ and metabolites.^20^ CNNs are a class of deep learning models that can easily process image data by using convolutional layers to automatically learn spatially correlated patterns. CNNs have also been used as a main framework for the automatic materials characterization of IR spectra.^2^ Recent approaches in NMR-based structure elucidation also include applying transformer-based ML models that have been pretrained on synthetic NMR spectra.^21^ This study also explored how performance of the language models can be improved if provided with ancillary chemical information such as the reagents and products of a given reaction along with each NMR spectrum. Another study showed how one can calculate the electronic structure and NMR spectrum of a candidate molecule and use a Bayesian approach to relate an experimental NMR spectrum with the simulated data.^22^ These approaches track a more general increasing tendency of ML methods to include additional prior knowledge, such as chemical formulas and molecular fragments,^13,23–27^ into the encoding process of ML methods to assist in automating materials characterization. The need for this extra molecular information as input to the fully trained ML models constrains their utility since such chemical data may be limited, circumspect, or unknown.

In this paper, we will demonstrate an ML method that solely requires 1D experimental NMR spectra to automatically determine chemical substructures of materials. Our ML models were trained using the data set of experimental ^13^C and ^1^H NMR spectra obtained from the open database nmrshiftdb2,^28,29^ which contains metadata about the environmental conditions of a given NMR experiment, such as field strength, temperature, and the solvent in which the NMR sample was dissolved. Knowledge of these conditions is crucial given that their nature affects the values of NMR chemical shifts. The training data for our ML models that interpret ^13^C NMR spectra also included data from the nmrshiftdb2 database that specify the type of *J–J* coupling constants for each unique carbon atom, which provides information about its chemical environment as an index, i.e., the type of peak splitting (multiplicity): singlet, s (1), doublet, d (2), triplet, t (3), and quaternary, q (4). Our findings will underscore the importance of including these experimental conditions and coupling-constant proxy measures as metadata. Our ML models embed spectrum–structure correlations into their neural-network architecture, wherein we explore the use of two methods for representing the molecular substructure, namely, the neighbor approach that focuses on identifying neighboring atoms and bond types and the other one being the widely used functional group approach. We compare the performance of three different ML models that are all based on one or more type of neural network: multilayer perceptron (MLP), long short-term memory (LSTM), CNN, and RNN. We also highlight the trade-off between accuracy and computational efficiency between these models and suggest practical alternatives for faster processing in real-world applications.

## Methods

### Data Curation

In this study, we used nmrshiftdb2, an open-access database that contains a comprehensive collection of experimental and computationally simulated NMR data.^29^ It allows users to download text files that detail key chemical information such as chemical table (CTAB) entries; the hashed, compact representations of International Chemical Identifier (InChI)—InChIKeys; chemical names; and coupling constants for each peak of an NMR spectrum. It also contains data that describe the experimental conditions under which the spectra were obtained, including the temperature, magnetic field strength of the NMR experiment, and the solvent used to dissolve the chemical sample to perform the solution NMR experiment. This information is not available for every chemical compound in the database, as the original metrological sources may not have reported all experimental conditions. The text files contain data for both experimental and computational spectra, and each spectrum is assigned a unique number. We only used experimental spectra for training our ML models as computational data may not accurately simulate real-world conditions, such as impurities and instrument-specific artifacts, while they may contain biases owing to theoretical limits of their underlying models or parameters used to generate such spectra. The experimental data were exclusively extracted from the database files by filtering out all of its “Program” data (meaning computational spectra).

We reconstructed spectral images from the nmrshiftdb2 database using chemical shift entries of experimental spectra given in the (input data) text files. The reconstruction step was chosen instead of using existing spectral images in the database because NMR spectral peaks are typically distinct and such data were readily available. Although we sourced a large number of experimental ^13^C and ^1^H NMR spectra from the nmrshiftdb2 database, a much smaller subset of ^13^C and ^1^H NMR spectra featured metadata about all three experimental conditions: temperature, field strength, and solvent. Overall, we considered 34,503 experimental ^13^C NMR spectra corresponding to 32,562 unique chemical compounds. These spectra contained a total of 361,823 chemical shift values. However, only 2086 ^13^C NMR spectra for 1978 compounds contained metadata that fully specified the three experimental conditions, temperature, field strength, and solvent; these totaled 34,563 chemical shift values. In the case of proton NMR, the nmrshiftdb2 data set contained 17,311 experimental ^1^H NMR spectra with a total of 123,976 chemical shift values. Only 987 of these ^1^H NMR spectra included all three experimental conditions: temperature, field strength, and solvent; these spectra totaled 6865 chemical shift values.

### Achieving Data Readiness for Input to ML Models

The original nmrshiftdb2 database text file contains chemical shift entries corresponding individually to each carbon (C) or hydrogen (H) atom within a molecule, derived from either ^13^C or ^1^H NMR experimental spectra. These individual chemical shift values served as input for our ML models. Given the significant influence of the chemical environment on chemical shift, the ML models were designed to recognize atoms within the same molecule and distinguish them from those in other molecules. This was achieved by representing the intramolecular connectivity of multiple atoms through an additional input. This input contains all chemical shift values of atoms within the same molecule. Furthermore, we also systematically ordered the input data from the smallest to largest chemical shift values.

The ^13^C NMR data from the nmrshiftdb2 database also includes categorical labels. These labels classify *J*–*J* coupling constants according to the interaction between probed carbon atoms and any neighboring hydrogen atoms in the immediate chemical environment. The categorical label is based on multiplicity classifications that indicate the number of hydrogen (H) atoms bonded to a given carbon atom. These classifications are categorized as 1 (no attached H), 2 (one attached H), 3 (two attached Hs), and 4 (three attached Hs). Based on the above categories, both coupling-constant classifications for individual carbon atoms and the complete set of coupling-constant indices for each molecule were included as two additional data input categories. To maintain consistency with the ordering of chemical shift values, the coupling-constant classifications were also ordered from the lowest to highest chemical shift values.

The nmrshiftdb2 database does not contain any data about NMR peak intensities. While this omission did not adversely impact the ML-based ^13^C NMR spectral characterization, it did have a compromising effect on the ^1^H NMR data, as our results herein will demonstrate.

Given the fact that chemical shifts in NMR spectra can be influenced by experimental conditions, we included field strengths, temperature, and solvents as input data for training the ML models, as summarized in Table 1. Further, we normalized each temperature and field strength value to their maximum values and assigned numerical categories to solvents using a min/max normalization given as

$$\mathrm{normalized}\;\mathrm{value}=\frac{\mathrm{original}\;\mathrm{value}}{\mathrm{maximum}\;\mathrm{value}}$$

where value refers to the temperature or the field strength and the normalized values range between 0 and 1.

**Table 1:** Example Entry of Input Data for the ML Models

input categories	input data before processing	input data after processing
field strength	50	0.07143
temperature	298	0.9226
Solvent	chloroform-*D*_1_ (CDCl_3_)	4
individual chemical shift	17.6	17.6
individual multiplicity	q	4
list of chemical shifts of atoms within the same molecule	17.6, 18.3, 22.6, 26.5, 31.7, 33.5, 33.5, 41.8, 42.0, 42.2, 78.34, 140.99, 158.3, 193.4, 203.0	17.6, 18.3, 22.6, 26.5, 31.7, 33.5, 33.5, 41.8, 42.0, 42.2, 78.34, 140.99, 158.3, 193.4, 203.0
list of multiplicities of atoms within the same molecule	q, t, q, t, t, s, s, t, s, d, s, s, d, d, d	4, 3, 4, 3, 3, 1, 1, 3, 1, 2, 1, 1, 2, 2, 2

Unavailable experimental conditions were set to zero during input data preparation. We also compared the performance of one-hot encoding versus integer encoding for categorical solvent data. The resulting accuracies were consistent between these two encoding methods.

Table 2 illustrates the extent to which the ^13^C NMR data set lacks metadata regarding these three experimental conditions. Approximately 94% of entries in the raw data file lacked metadata about at least one of the three experimental conditions: field strength, temperature, or solvent. Moreover, 89% lacked metadata for all three conditions. To address these issues, we designed and employed a statistical imputation method to recover some of the missing metadata by utilizing common chemical features among spectra. The imputation method was developed in three stages. First, we identified and selected common functional groups that were present in data sets with and without specified experimental conditions. Second, we contrasted the values of chemical shifts in data entries containing specified conditions against those from entries lacking such metadata. Finally, we attempted to estimate the unknown experimental conditions by mapping these chemical shift values of the entries with missing metadata to those with known metadata. Despite these efforts, the task proved challenging. In most cases, the data entries with missing metadata lacked information for two or more of the three experimental conditions. Therefore, it was difficult to isolate the effects of each individual condition (temperature, field strength, and solvent) on the chemical shift values. Due to this limitation, we decided to retain the raw data without applying data imputation.

**Table 2:** Quantity of ^13^C NMR Data that Lack Metadata about Their Experimental Conditions, Temperature, Field Strength, and Solvent

experimental conditions for which metadata are missing	count
field strengths	31,558
solvents	30,690
temperatures	31,735
field strengths and solvents	31
field strengths and temperatures	235
solvents and temperatures	16
all conditions	30,642

### Representing NMR Spectrum–Structure Correlations within a Neural Network

There are several distinct ways to identify chemical substructures of a material from spectrum–structure correlations.^2,11,12,23^ We identified two main approaches: an atomic neighbor approach and a functional group approach. The atomic neighbor approach characterizes the local environment by considering the four possible bonds formed by a carbon atom in a molecule during ^13^C NMR spectral analysis. A previous study used graph-theoretical methods to denote atoms and bonds as vertices and edges, respectively, to model molecular connectivity.^23^ In this study, we propose a new type of atomic neighbor approach for encoding and identifying chemical substructures in organic molecules. The generic neural network architecture developed for this neighbor approach is illustrated in Figure 1a, with specific details explained in the following subsection. This architecture was embedded into each type of ML model used in this study, and their resulting predictive abilities were evaluated and compared.

**Figure 1:**
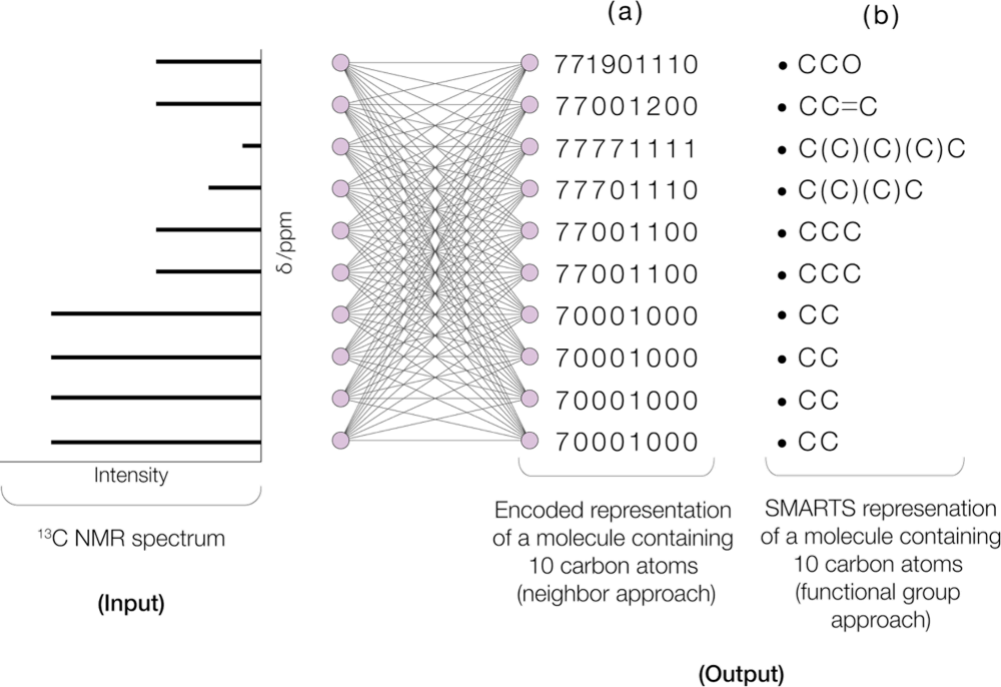
Schematic representation of the ^13^C NMR spectral input to the neural-network architectures in this study and their output according to whether or not spectrum–structure correlations are represented by the (a) neighbor approach or (b) functional group approach.

The functional group approach,^2,7,11,12^ commonly adopted in structure elucidation, involves identifying a group of atoms or bonds connected in a specific way within a molecule. Functional groups such as alkenes, ketones, and carboxylic acids retain their physical and chemical properties irrespective of their host compound. Accordingly, recognizing these characteristic properties of constituent functional groups offers a way for inferring the structure of an unknown compound. In this study, we incorporated the functional group approach for each ML model using a generic neural-network architecture shown in Figure 1b. The predictive capabilities of these ML models were compared against those employing our novel atomic neighbor approach.

#### Neighbor Approach

This approach was based on the information extracted from CTAB entries in the raw data file, which specify the neighboring atoms and bond types that are connected to each carbon atom. In total, we identified 31 atom types (e.g., Ag, F, and Ti, in Table 3) and bond types that ranged from nonexistent to triple bonds. For each carbon atom, the local environment was encoded using a set of eight output labels (see Figure 2). Each of the first four numerical labels denoted an atom type bonded to the target carbon atom, indexed numerically based on alphabetical order as indicated in Table 3. If a carbon atom was bonded to fewer than four atoms, then the unused positions were assigned a value of 0. The remaining four labels denote the corresponding bond types with values ranging from 0 (no bond) to 3 (triple bond). The last four labels were mapped element-wise to the first four labels. For example, if the third label is 0, then the seventh label should also match 0. Due to the incomplete record of hydrogen atoms in the CTAB entries, a postprocessing step was needed to ensure consistency. For instance, following the initial round of labeling, some atoms had their last four labels adding up to 4, while others did not, indicating inconsistencies. Therefore, if any of the first four labels represented hydrogen, both the atom and associated bond types were reset to zero. For this reason, the last four labels only add up to 3 in the example shown in Figure 2. A complete list of substructure labels used in the neighbor approach is provided in the Supporting Information (SI. 1).

**Figure 2:**
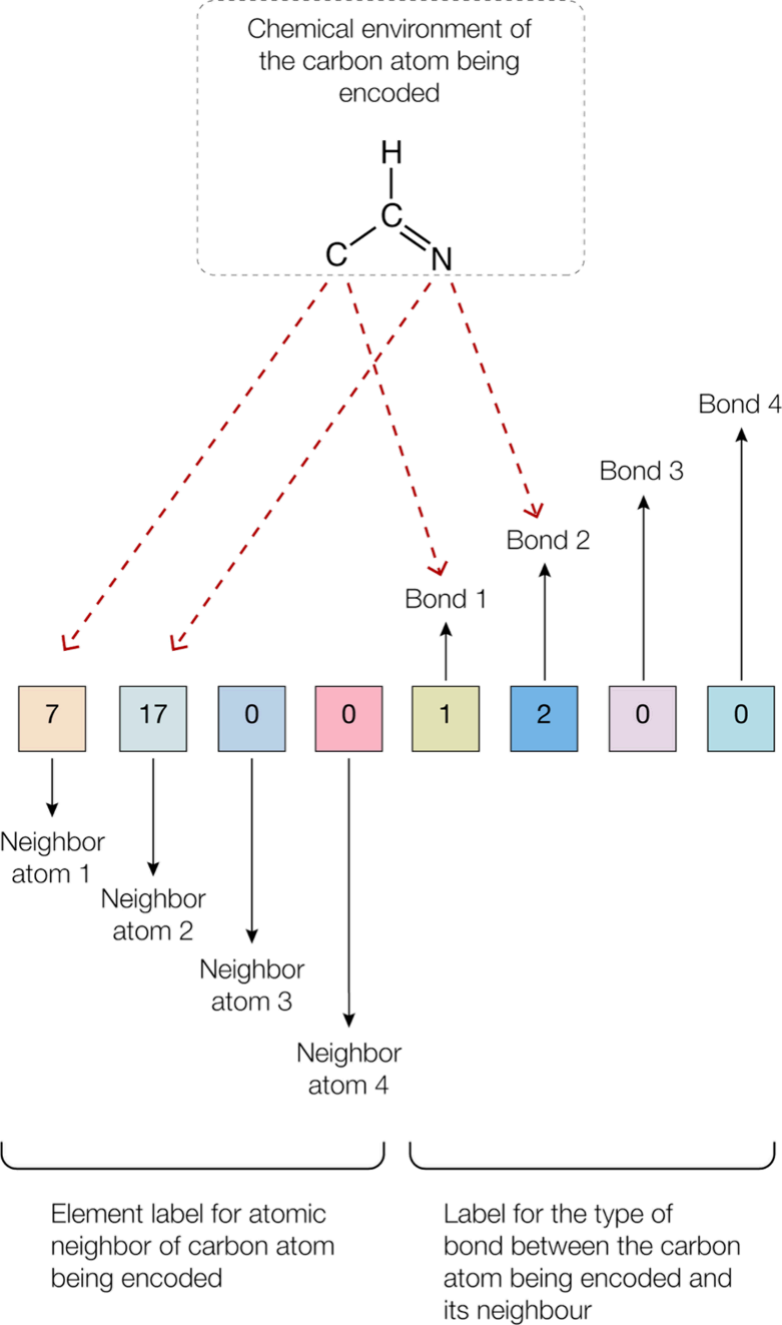
Representation of the eight output labels of the neighbor approach where the first four correspond to the numerical indices (0–31) for the elements of the atoms that are chemically bonded to the carbon atom being encoded (see Table 3); the last four denote the order of the bond that chemically connects the carbon being encoded to its neighbor (1–3 for single, double, and triple bonds; 0 if the bond is to a hydrogen atom). In the example given, the numerical entries for the last four labels do not add up to four due to implicit hydrogen bonds. The sequential ordering of the neighbor atoms within each quadrant does not matter after sorting as long as the order of the interquadrant pairs is maintained, i.e., label 7, 17, 0, 0, 1, 2, 0, 0 is the same as 17, 7, 0, 0, 2, 1, 0, 0.

**Table 3:** Numerical Labels for Each Elemental Class and One Exception (for Class Number 32)

atom type	numerical label	atom type	numerical label
Ag	1	N	17
Al	2	Na	18
As	3	O	19
B	4	P	20
Bi	5	Pb	21
Br	6	Pd	22
C	7	S	23
Cl	8	Sb	24
F	9	Se	25
Ge	10	Si	26
H	11	Sn	27
Hg	12	Te	28
I	13	Ti	29
K	14	Tl	30
Li	15	Zn	31
Mg	16	except for C, N, and O	32

Each of the first four labels has 32 elemental classes, as defined in Table 3, while each of the last four labels encoded one of four bond-type classes. To ensure consistency, the four bond-type labels were sorted in ascending numerical order, as shown in the example given in Figure 2. In cases where multiple bond-type labels shared the same value, they were further sorted based on the categorical values of their associated atom-type labels. For instance, a label sequence consisting of 7, 7, 19, 17, 1, 1, 1, 1 would be reordered as 7, 7, 17, 19, 1, 1, 1, 1. Initially, the molecular substructure was defined by a combination of eight labels, and the outputs were assigned single categorical values based on their label combinations. Identical label sets were consolidated into a single label. Although integer coding could be used, it allows the ML models to assume a natural ordering between the categories, leading to poor model performance. We avoided this issue by employing one-hot encoding to represent these eight single labels. Each one-hot encoded label was shortened, and all nonzero categorical values, except those representing carbon (C), oxygen (O), and nitrogen (N), were assigned the same categorical value. This process reduced the label from 503 to 136 values for the complete data set.

For the ^1^H NMR training data set, each hydrogen atom with chemical shift data was attached to a carbon atom. Consistent with the method described above for classifying ^13^C NMR spectral data, we took similar steps to classify the neighbors of the attached carbon atom as the substructure of the hydrogen atom. The atom types and their corresponding numerical labels used for the ^1^H NMR spectral data are provided in the Supporting Information (SI. 1).

#### Functional Group Approach

As mentioned earlier, the functional group approach is one of the most widely used approaches for defining molecular substructures. In this study, we generated molecular objects from the CTAB entries using the open-source toolkit RDKit^30^ while preserving the original atom numbering given in the raw file. This step enabled a mapping between atoms with their corresponding chemical shift values. Following this step, the implemented code identified atoms within each molecule that belonged to specific functional groups. Further, our substructure elucidation framework employed a derivation of the chemical notation system, SMILES (Simplified Molecular Input Line Entry System), which encodes chemical structures as machine-readable text strings.^31^ This derivation is known as SMARTS (SMiles ARbitrary Target Specification strings); a SMARTS string represents a chemical substructure and is created by extending a SMILES string using a rule-based approach.^32^

To define the functional groups of interest, we initially compiled a list of SMARTS strings (Table 4) from common knowledge and various journal papers. However, this initial list was not exhaustive, and certain atoms were not associated with any functional group on the list. Therefore, we manually identified additional functional groups via examination of individual atom structure diagrams and appended them to the list. Such an examination ensured a comprehensive classification of each atom. Later, these functional groups were represented by categorical values and then one-hot encoded to generate output labels. Since the code searched through the list of the functional groups for each atom in a molecule and assigned it to one functional group only, the order of the list influenced the model performance. Moreover, longer SMARTS strings tend to capture more characteristics within a functional group, and such a well-defined functional group typically leads to a better classification. First, the list of SMARTS strings was arranged in a manner where aromatic molecules preceded aliphatic groups. Within each group, strings were sorted by length from longest to shortest. This initial ordering method served as a baseline for subsequent testing. It was reassessed after establishing a functional model. Following 100 random reorganizations of the list, the order yielding the highest model accuracy afforded the optimized arrangement. The randomization resulted in an approximately 5% accuracy improvement for the complete data set of ^13^C NMR spectra using the functional group approach and the CNN model architecture.

**Table 4:** SMARTS Definitions for the Functional Groups Used for ^13^C NMR Data with All Three Experimental Conditions Specified^b^

definition	SMARTS^a^
sulfoxide	[#6][#16X3]=[OX1]
carbamate	[NX3][CX3](=[OX1])[OX2]
sulfonamide	[#16X4]([NX3])(=[OX1])(=[OX1])[#6]
	[#6;R][#6;R]
	[#6;R]=,:[#6;R]
hydrazone	[NX3][NX2]=[#6]
ether	[OD2]([#6])[#6]
amide	[NX3][CX3](=[OX1])[#6]
thiol	[#6][#16X2]
	[CX4H2]
	[CX4H0]
benzyl	[CX4][cX3]1[cX3][cX3][cX3][cX3][cX3]1
	[#6;R][#7;R]
alkene	[CX3]=[CX3]
sulfonate	[#16X4](=[OX1])(=[OX1])([#6])[OX2]
haloalkane	[#6][F,Cl,Br,I]
	[CX4H3]
imine	$([CX3]([#6])[#6]),
	$([CX3H][#6])]=[$([NX2][#6]),$([NX2H])]
	[N][C][N]
phosphine	[#6][PX3]
	[C](=C)(=C)
nitrile	[NX1]#[CX2]
	[CX3](=O)[OX2H1]
aldehyde	[CX3H1](=O)[#6]
alkyne	[CX2]#[CX2]
thioamide	[NX3][CX3]=[SX1]
ester	[#6][CX3](=O)[OX2H0][#6]
	[CX4H1]
	[C](=N)
carbonyl	[CX3]=[OX1]

a Not all patterns presented by the SMARTS strings have a clear functional group definition.

b The order of the list presented here corresponds to that which was obtained after optimization.

Each hydrogen atom with chemical shift data from the ^1^H NMR spectra was linked to a carbon atom through a chemical bond. Therefore, a similar procedure was followed to derive SMARTS strings for the ^13^C NMR spectra data and extended to classify the functional group, whereby this assigned carbon atom became the substructure of the hydrogen atom. The SMARTS definitions for the functional groups used for the ^1^H NMR spectral data can be found in Table 5.

**Table 5:** SMARTS Definitions for the Functional Groups Used for ^1^H NMR Data with All Three Experimental Conditions Specified^a^

SMARTS
[CX2]#[CX2]
[CX3]=[P]
[CX3][CX2]
[#16X4]([NX3])(=[OX1])(=[OX1])[#6]
[CX4H3]
[#6;R][#6;R]
[#6;R]=[#7;R]
[CX3](=O)[OX2H1]
[#6;R]=,:[#6;R]
[NX3][CX3]=[CX3]
[NX3][CX3](=[OX1])[#6]
[CX3](=[OX1])[OX2][CX3](=[OX1])
[#6;R]=S
[CX3]=[OX1]
[CX3H1](=O)[#6]
[NX3][NX2]=[#6]
[#16X4](=[OX1])(=[OX1])([#6])[#6]
[N][C][N]
[NX1]#[CX2]
[NX3][CX3](=[OX1])[OX2]
[#6]1:[#6]:[#7]:[#7]:[#7]:1
[CX3]=[Se,S]
[#6][CX3](=O)[OX2H0][#6]
[CX3]=[CX2]
[#6;R]=[O;R0]
[CX4][cX3]1[cX3][cX3][cX3][cX3][cX3]1
[#6][NX3][NX3]
[OX2H][#6X3]=[#6]
[#6;R][#7;R]
[#6]1[#6][#6][#6][#6][#6]1
[CX4H4]
[CX3](=[OX1])[NX3][CX3](=[OX1])
[CX3]=[CX3]
[C](=N)
[#6][PX3]
[#6][#16X2]
[CX1-]
[#6][NX2]=[NX2][#6]
[#6][CX3](=O)[#6]
[$([CX3]([#6])[#6]),$([CX3H][#6])]=[$([NX2][#6]),$([NX2H])]
[cX3]1[cX3][cX3][cX3][cX3][cX3]1
[#6][OX2H]
[CX4H2]
[#6][F,Cl,Br,I]
[CX3-]
[CX2]=[OX1]
[NX3][CX3]=[SX1]
[CX4H0]
[#6;R]=[#6;R0]
[OD2]([#6])[#6]
[CX3+]
[CX1]#[NX2]
[CX4H1]
[C](=C)(=C)
[CX3](=[OX1])[F,Cl,Br,I]
[CX3]=[SX1]
[#6][#16X3]=[OX1]
[CX3]=[SX2]
[#6;R][#8;R]
[#16X4](=[OX1])(=[OX1])([#6])[OX2]
[#6;R]=[Se]
[OX2H][cX3]1[cX3][cX3][cX3][cX3][cX3]1

a The order of the list presented here corresponds to that which was obtained after optimization.

### ML Model Training

#### ML Model Design Considerations

The four types of neural-network architectures employed in this study have specific attributes that make them potentially well-suited for the subject task. MLPs are effective in tackling spectral data as they perform well with vectors and arrays, and they are useful for classification tasks.^33^ CNNs are best for handling image data as they can automatically learn spatial hierarchies of features, making them highly effective for image classification and object detection.^34^ While an MLP uses weighted additions, a CNN uses convolution layers for feature extraction. Thus, CNNs have reduced connectivity and complexity compared to MLPs but maintain model performance. However, CNNs fall short in handling sequential data and so, where order matters, RNNs become a possible option. RNNs maintain memory via recurrent connections and are particularly effective for processing sequential data.^4,35^ However, RNNs do not perform well if the length of sequences is increased as the model gradually loses initial information about the initial inputs in the later recurrent units. This happens because of vanishing and exploding gradient issues, which are addressed by the alternative use of long short-term memory (LSTM) networks. LSTM^35^ is a variant of RNN that employs gating mechanisms to regulate the flow of information throughout the network, thereby enabling them to capture long-term dependencies more effectively. In summary, MLPs handle well vectors and arrays, CNNs excel in extracting spatial information, RNNs tackle sequential data, and LSTMs enhance the capacity of RNNs by accommodating long-term dependency learnings.

Therefore, the ML model designs explored in this work for elucidating the chemical substructures of NMR spectra centered on the use of three different neural-network architectures: a CNN model given that NMR spectra are inherently shaped by spatially correlated data points and an MLP model that was combined with either an RNN or LSTM network (LSTM + MLP and RNN + MLP) given that NMR spectra are produced from sequential data vectors or arrays, the modeling of which may benefit from long-term dependency learnings. We also implemented into our ML model design an assessment of the relative impact of representing NMR spectra via either the neighbor approach or the functional group approach on ML model performance. Furthermore, we tested how the accuracy of our ML models is affected by the inclusion of metadata from the aforementioned three experimental conditions.

#### ML Model Training Details

First, we trained neural networks only on data with available experimental conditions and chemical shift values for individual atoms. These models were then trained on all data, including those with missing experimental conditions. Considering the significance of the chemical environment in determining chemical shifts of NMR spectra, we added the complete list of chemical shifts for all atoms in the same molecule as an extra input.

#### RNN + MLP Model

Given the aforementioned strength of RNNs on handling sequential input data, we selected RNNs to process the list of chemical shifts whose data exhibited variable lengths. All data of the same length were alternatively fed into the input of an MLP. The output from these two networks was concatenated and passed through additional feed-forward neural network layers before predicting the final output labels.

#### LSTM + MLP Model

The RNN layers of the aforementioned neural network architecture were then replaced with LSTM layers, thereby affording an MLP + LSTM network. The variable input was passed into two LSTM layers in both directions, as depicted in Figure 3. Specifically, 50 hidden states from 50 cells from the forward and backward layers were concatenated and became the input layer for two MLP layers. Then, the fixed input was concatenated with the 500 nodes from the second set of MLP layers.

**Figure 3:**
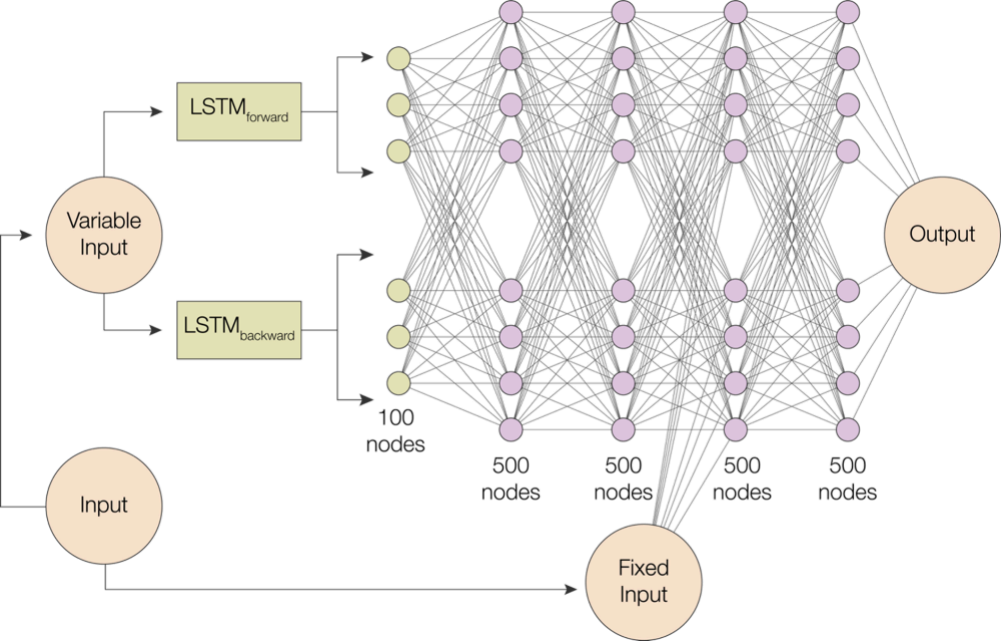
Illustration of the MLP + LSTM model architecture used in this study. The input variable was passed through two bidirectional LSTM layers, with 50 hidden states concatenated from both forward and backward directions. These were then used as the input to two MLP layers, followed by concatenation with the fixed input from the second MLP layer, which contained 500 nodes.

#### CNN Model

The prospective utility of the CNN architecture shown in Figure 4 for this work was then explored. Zero padding was employed to account for the typical fixed-size input of a CNN design. The layers of its neural networks were formulated via trial and error. In the first instance, the functionality of our CNN model was checked by randomly selecting two molecules as test samples and manually inspecting the processed CNN output. For its full model training, we split the data set into two subsets: 20% for testing and 80% for training. We performed 3-fold cross-validation to improve the validity of metric evaluations.

**Figure 4:**
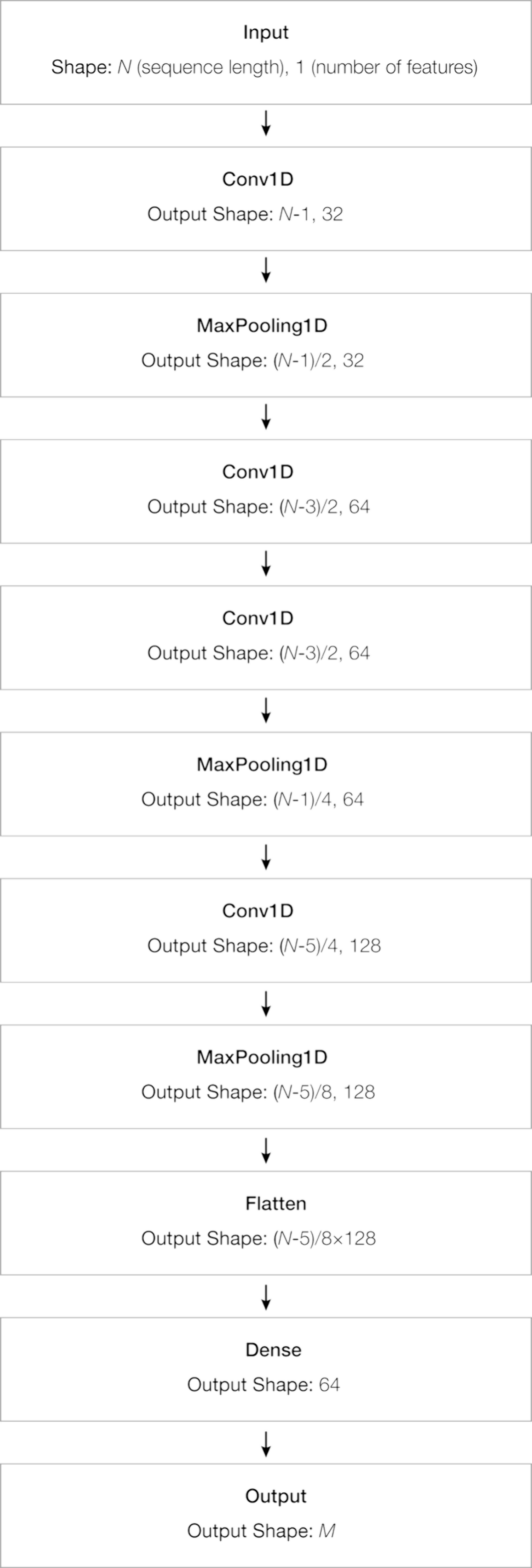
Schematic of the CNN architecture used in this work. The way in which data were divided by the MaxPooling1D layer varied for different output data sets; this figure illustrates the case where the data set was divided by 2. The input data set size was labeled as *N*, and the output data set size was *M*.

#### Hyperparameter Tuning of ML Models

We employed cross-validation using the GridSearch option provided in scikit-learn^36^ to optimize hyperparameters of the neural network models provided by Keras.^37^ We optimized the following hyperparameters: (i) number of layers of the model, (ii) batch size and training epochs, (iii) optimization algorithms, (iv) learning rate and momentum, and (v) neuron activation functions in hidden layers of the model.

The number of layers of models was determined manually, starting with a moderate number of layers. Then, we incrementally increased this number until its validation accuracy hit the point of diminishing returns. The optimum batch size and number of epochs for training were determined by tracking model accuracy as a function of the number of epochs (up to 200 epochs) during the cross-validation exercise, for a given batch size that was varied from 30 to 6000. The results indicate that an appropriate number of epochs lies between 100 and 150; for example, see results for the MLP + LSTM model in Figure 5.

**Figure 5:**
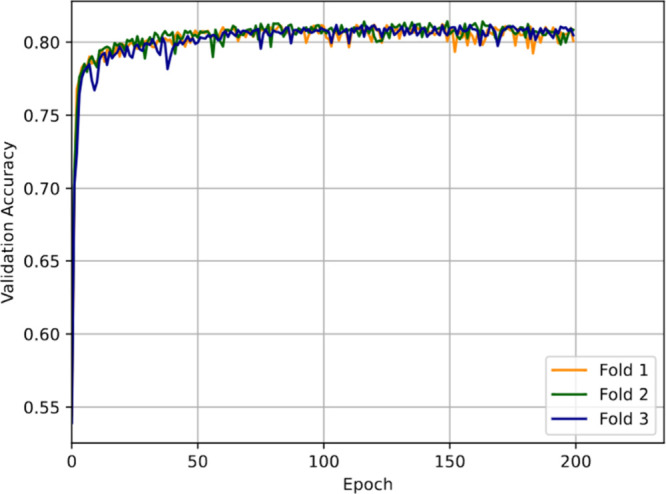
Validation accuracy of the MLP + LSTM model as a function of the number of training epochs assessed over a process of three-fold cross-validation. The validation accuracies plateaued after 50 epochs and peaked between 100 and 150.

Stochastic gradient descent (SGD), Adadelta, AdaGrad, RMSprop, Adam, AdaMax, and Nadam optimization algorithms from the Tensorflow^38^ platform were each deployed in a grid search to fine-tune the optimization algorithm. The optimum learning rate constituted the best trade-off between precision and efficiency, and this was found by performing a grid search for different parameter values for the various ML models and inputs. The momentum of the “Adam” (adaptive moment estimation) optimizer^39^ was sampled between 0 and 1 with an increment of 0.2, to determine the optimum parameter values for each ML model. Adam-like optimizers were found to be the most appropriate for the data set, which stands to reason given their balanced approach of adaptive learning rates, diminishing gradients, and incorporating momentum. Plots of model accuracy and model loss for the learning rate and momentum parameters (Supporting Information, SI. 3, Figure S3.1) showed considerable fluctuations even though their final training and validation accuracies were high (see Figure 5). Models using the default learning rate and momentum values exhibited more stable training and validation histories. Therefore, the default values were chosen for the final results.

Softmax activation functions for the MLP + LSTM and MLP + RNN models were fine-tuned by comparing the validation accuracies manually, given that these models are concerted.

The optimum hyperparameters used in the final ML models are provided in the Supporting Information, SI. 2 (Tables S2.56–64). Training and validation history of the accuracy and loss for all final ML models as a function of the number of epochs are given in the Supporting Information, SI. 3. These model histories of accuracies and losses were also used to check for overfitting.

NVIDIA GeForce RTX 4080 hardware was employed for these tasks. Thereby, timing data differ according to whether or not a GridSearch option was used and how many parameters were involved in the optimization process. For example, the timing data ranged from 0.5 to 1.5 days if a GridSearch was engaged with the ANN + LSTM model; meanwhile, it required ∼3 h to train the ANN + LSTM for the complete data set and ∼1 h for CNN, using optimized parameters.

### ML Model Performance Results

#### Evaluation Metrics

The performance of each ML model was captured by standard evaluation metrics that are calculated based on an *N* × *N* array of nested 2 × 2 confusion matrices whose elements denote true positive (TP), true negative (TN), false positive (FP), and false negative (FN). TP and TN refer to the number of cases where a model correctly predicts a positive or negative classification, respectively; FP and FN are defined as the number of times the model incorrectly predicts a positive or negative classification, respectively. These elements define the evaluation metric for accuracy, which is given mathematically as

$$\mathrm{accuracy}=\frac{\mathrm{TP}+\mathrm{TN}}{\mathrm{TP}+\mathrm{TN}+\mathrm{FP}+\mathrm{FN}}$$

where accuracy measures the ratio of correctly predicted labels to the total number of labels for a given binary classification.

Their associated confusion matrices form nested 2 × 2 matrices within a larger *N* × *N* array because the output labels from our ML models are all one-hot encoded; so, the true and prediction labels are lists of lists. The *N* × *N* confusion matrix was constructed by the process shown in Figure 6. Thereby, the list of lists was first converted into flat lists of class labels. The index of elements with the largest probability was used for a flat list of prediction labels. Likewise, the index of elements with values equal to 1 was used for the flat list of true labels. The two resulting flattened lists contain *N* elements and form the *N* × *N* confusion matrix.

**Figure 6:**
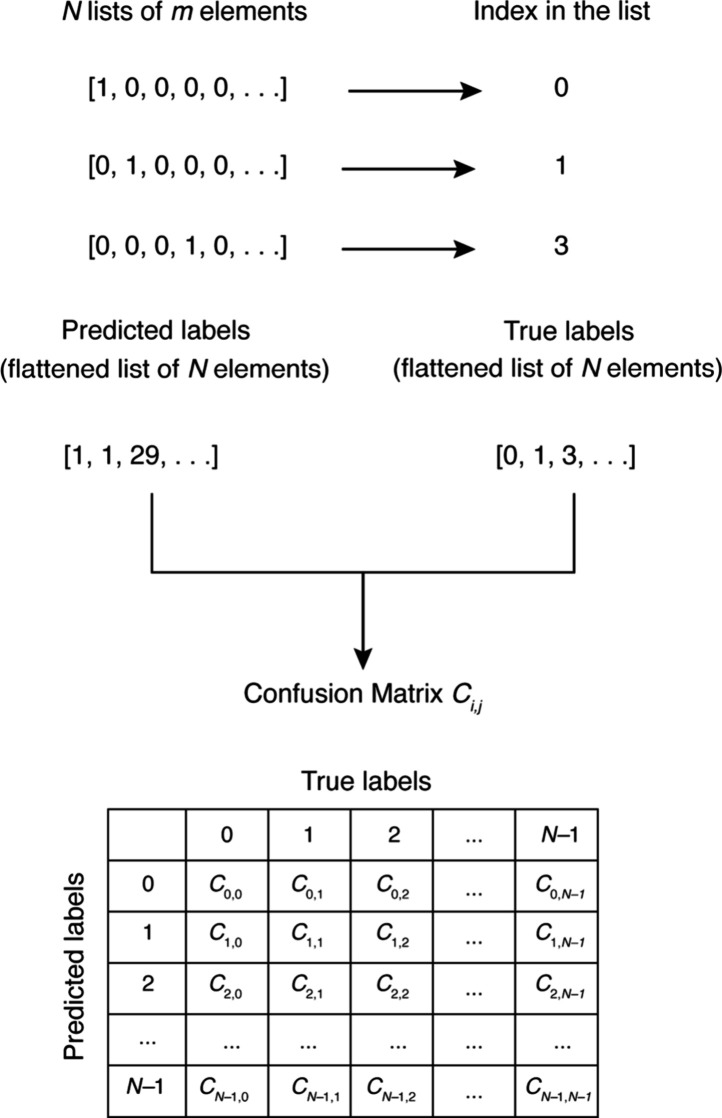
Each list of one-hot encoded labels contains *m* elements; the size of the output layer of the model is *m*. The flattened lists have *N* elements where *N* corresponds to the number of testing data, also denoting the number of classes for this confusion matrix. The resulting confusion matrix, *C*_*i,j*_, where 0 ≤ *i*, *j* ≤ *N –* 1 yields relevant evaluation metrics. Note that the sum of all entries in the matrix *C*_*i,j*_ is *N*.

Therefore, the value of each element within each confusion matrix, *C*_*i,j*_, can be computed for each class *i* where 0 ≤ *i* ≤ *N* – 1. TP*_i_* represents the diagonal element of the confusion matrix, i.e., TP*_i_* = *C*_*i,i*_. Similarly, FP*_i_* is the sum of *i*th column minus the diagonal element *C*_*i,i*_ given as FP*_i_* = $\sum_{k=0}^{N-1}C_{k,i}‐C_{i,i}$ ; FN*_i_* = $\sum_{k=0}^{N-1}C_{i,k}‐C_{i,i}$ ; TN_*i*_ = *N* – (TP*_i_* + FP_*i*_ + FN*_i_*) where $N=\sum_{j=0}^{N-1}\sum_{k=0}^{N-1}C_{j,k}$ .

The overall accuracy of the entire matrix can then be given as

$${\mathrm{accuracy}}_{\mathrm{overall}}=\frac{\overset{N-1}{\underset{i=0}{\sum }}{\mathrm{TP}}_{i}}{N}$$

where *N* = $\sum_{i=0}^{N-1}{\mathrm{TP}}_{i}+{\mathrm{TN}}_{i}+{\mathrm{FP}}_{i}+{\mathrm{FN}}_{i}$ .

The overall precision, recall, and F1 score for the entire matrix can be microaveraged in the following ways:

$${\mathrm{precision}}_{\mathrm{micro}}=\frac{\sum_{i=0}^{N-1}{\mathrm{TP}}_{i}}{\sum_{i=0}^{N-1}{\mathrm{TP}}_{i}+{\mathrm{FP}}_{i}}$$

$${\mathrm{recall}}_{\mathrm{micro}}=\frac{\sum_{i=0}^{N-1}{\mathrm{TP}}_{i}}{\sum_{i=0}^{N-1}{\mathrm{TP}}_{i}+{\mathrm{FN}}_{i}}$$

$$F1‐{\mathrm{score}}_{\mathrm{micro}}=2\times \frac{{\mathrm{precision}}_{\mathrm{micro}}\times {\mathrm{recall}}_{\mathrm{micro}}}{{\mathrm{precision}}_{\mathrm{micro}}\times {\mathrm{recall}}_{\mathrm{micro}}}$$

where microaveraging implies that more weights are given to classes with more instances. On the other hand, macroaveraging treats each class equally. The classes of substructures used in this paper were imbalanced because some substructures occurred more often than others. Therefore, it was necessary to employ microaveraged metrics for the evaluation in the paper.

For one-hot encoded labels, each instance is exclusively assigned to one class. Any error made in predicting one class corresponds directly to an error in predicting another class. Therefore, the microaveraged metrics carry the same value as the accuracy.

#### Comparison of Resulting ML Models

Overall, a full set of MLP + RNN, MLP + LSTM, and CNN models was developed that use the neighbor approach and that were trained on either the full set of ^13^C NMR spectral data (complete data) or its data subset where all three experimental conditions are specified (clean data). The accuracy metrics for the MLP + RNN models were consistently lower than those of the MLP + LSTM models (see Table 6). This poorer model performance originated from a vanishing gradient problem within the RNN architecture. Therefore, MLP + RNN models were consequently excluded from further consideration.

**Table 6:** Accuracy Results for the Neighbor Approach Using ^13^C NMR Spectral Data

model	accuracy using complete data (%)	accuracy using clean data (%)	accuracy using unspecified data (%)
MLP + LSTM	81	88	77
CNN	81	86	77
MLP + RNN	78	82	N/A

The MLP + LSTM and CNN models were also trained on a subset of the ^13^C NMR spectral data whose size was akin to that of the clean data set but which had its experimental conditions unspecified; this enabled a direct comparison of the effect of specifying experimental conditions where all three are present. The accuracy metrics for all ML models trained on data with unspecified experimental conditions are considerably lower than analogous ML models that were trained using the clean data where all three experimental conditions are specified. This result shows that specifying experimental conditions is crucial for improving ML model performance.

The MLP + LSTM and CNN models were then trained on the complete data set and clean data set of ^13^C NMR spectra using the functional group approach. A comparison of Tables 6 and 7 indicates that ML models trained using the functional group approach will predict chemical substructures with a higher accuracy than the neighbor approach.

**Table 7:** Accuracy Results for the Functional Group Approach Using ^13^C NMR Spectral Data

model	accuracy using complete data (%)	accuracy using clean data (%)
MLP + LSTM	84	86
CNN	86	86

The analogous comparison was then made for ML models that we trained on ^1^H NMR spectra using the neighbor approach (Table 8) or the functional group approach (Table 9). These results corroborate the notion that ML models trained using the functional group approach are superior to those trained using the neighbor approach.

**Table 8:** Accuracy Results for the Neighbor Approach Using ^1^H NMR Spectral Data

model	accuracy using complete data (%)	accuracy using clean data (%)
MLP + LSTM	63	69
CNN	60	66

**Table 9:** Accuracy Results for the Functional Group Approach Using ^1^H NMR Spectral Data

model	accuracy using complete data (%)	accuracy using clean data (%)
MLP + LSTM	74	76
CNN	71	75

The overall accuracy metrics for the ML models trained on ^1^H NMR spectra are significantly lower for those trained on ^13^C NMR spectra (Table 8 versus Table 9). This stands to reason because ^1^H NMR spectral peak intensities are not recorded in the nmrshiftdb2 database and so this information cannot be incorporated into the ML model during its training.

Overall, MLP + LSTM models performed slightly better than CNN models, irrespective of the approach used or whether or not they were trained on ^13^C or ^1^H NMR spectra, as judged by the accuracy metrics in Tables 6–9. However, it is worth noting that the CNN models are significantly more computationally efficient than the MLP + LSTM models. Thereby, the average run time of code executing a CNN model is about one-third that of the corresponding MLP + LSTM model. Given that the performance accuracy of CNN models is only slightly lower than that of MLP + LSTM models, other performance criteria should also be borne in mind. In particular, the good performance speed of a CNN model will foster widespread utility, while energy sustainability is also an important consideration. As such, we advocate that the CNN model be considered as a more practical choice for an automated materials characterization tool for ^13^C and ^1^H NMR spectra.

For Tables 6, 7, 8, and 9, the accuracy is quoted with an uncertainty of 2% considering the range of performance through randomizing the train/split cut.

### Demonstration of the Best-Performing CNN Model

#### Four Case Studies

The best-performing ML model to predict molecular substructures across all types of NMR data explored herein employs ^13^C NMR spectra with a CNN model using the neighbor approach to encode its spectrum–structure correlations (see Table 6). We now demonstrate the extent to which this ML model can successfully predict the molecular substructures of four chemical compounds solely from their ^13^C NMR data.

It is not viable to demonstrate the efficacy of this CNN model using a representative set of molecules since the field of organic chemistry is so vast. While the default option in such circumstances may be to select a random set of molecules for testing, we selected a more strategic option. Thereby, four compounds were selected based on (a) their practical utility across a diverse range of industrial applications, (b) their chemical diversity, and (c) their inclusion of chemically equivalent atoms, including intramolecular mirror symmetry, since that will affect the number of unique peaks within the ^13^C NMR spectra.

These four molecules are the plant-based health drug, beta-sitosterol; popular stimulant, caffeine; common painkiller, aspirin; and molecular building block, cyclopropylbenzene. Their chemical schematics are given in Figure 7.

**Figure 7:**
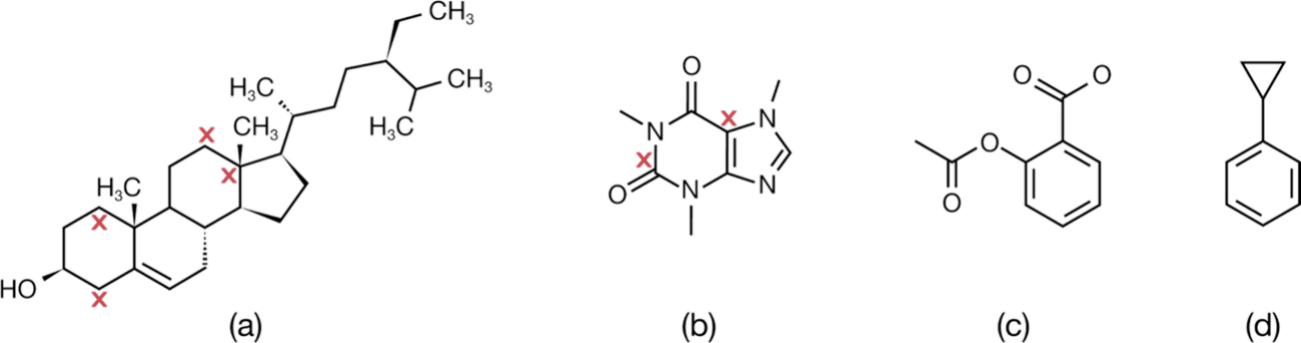
Chemical schematics of the four molecules in this case study: (a) beta-sitosterol, (b) caffeine, (c) aspirin, and (d) cyclopropylbenzene. The CNN model uses the neighbor approach to successfully predict 86.2, 72, 100, and 100% of all carbon atom substructures in (a–d) in terms of their immediate chemical environment, solely from unknown ^13^C NMR spectra. The red crosses show the carbon atoms whose surrounding substructures were not correctly predicted from their ^13^C NMR spectrum.

Table 6 shows that the overall model accuracy for the CNN model using the neighbor approach is 86%, where the field strength, temperature, and solvent used in the ^13^C NMR experiment were all specified as per cases (a–c) or 81% if not specified as per case (d). The predictions from these individual case studies are comparable to these overall model metrics, within a standard deviation of 14%. Indeed, all carbon atom substructures for aspirin and cyclopropylbenzene are correctly predicted from their ^13^C NMR spectra. The successful prediction for cyclopropylbenzene highlights the ability of the ML model to correctly assign molecular substructures even when chemically equivalent carbon atoms exist owing to its intermolecular mirror symmetry.

Caffeine displays the lowest accuracy (72%), although it should be borne in mind that caffeine only possesses eight carbon atoms; so, this result means that six out of eight carbon atom substructures are correctly predicted from its ^13^C NMR spectrum. Beta-sitosterol is a much larger molecule, affording a higher accuracy (86.2%), while four of its carbon atom substructures are incorrectly predicted from its ^13^C NMR spectrum. Full details of input data and output results for each case study are given in the Supporting Information, SI. 4, while we focus below on the salient findings.

Figure 7 highlights the incorrectly predicted carbon atom substructures for beta-sitosterol and caffeine, via red crosses, one of which is labeled against each affected carbon atom. One of the incorrectly predicted carbon atom substructures in beta-sitosterol is associated with a quaternary carbon atom. Its corresponding ^13^C NMR signal will therefore be very weak, which could make its prediction difficult. Moreover, the nmrshiftdb2 data that were used to train the CNN model will contain fewer quaternary carbon atoms in its repository of molecules relative to other types of carbon atoms, purely on the grounds of a geometric frequency argument. Thus, quaternary carbon atoms are less well-represented in the ML model training process, which stands to compromise the predictive abilities of such carbon atom substructures.

The other three incorrectly classified carbon atom substructures in beta-sitosterol all emanate from sp^2^ carbon atoms that lie within singly bonded, fused, six-member carbon rings. All their chemical shift values are significantly higher than those of the three other carbon atoms in beta-sitosterol that carry this same chemical classification, and yet, whose substructures are correctly predicted by the CNN model. This stands to reason because two of the carbon atoms involved in the incorrect substructure predictions are vicinal to a quaternary carbon, while the third carbon atom lies between a carbon involved in a C=C double bond and a carbon that bears a hydroxy group. By extension of the above geometric frequency argument, carbons that neighbor a quaternary carbon atom will be less represented in the training data of the ML model; similarly, carbon atoms that neighbor carbon atoms that bear heteroatomic substituents will be less represented in the ML training data than carbon atoms that are not structurally close to heteroatoms. The relatively lower representation of these types of sp^2^ carbon atoms within the training data could thus explain their lower predictive acuity.

Meanwhile, the two incorrectly predicted carbon substructures in caffeine correspond to two carbon atoms that are vicinal to at least one nitrogen atom. In both cases, the incorrectly predicted substructures differ from the ground truth by the model thinking that one of its atoms is a carbon when it is in fact a nitrogen atom. This mistake stands to reason given that the more carbon-based substructural motif that it incorrectly predicts will be more frequently represented in organic molecules (and thus the nmrshiftdb2 training data) than the nitrogen-bearing ground-truth substructure. Furthermore, both of the incorrectly predicted carbon substructures in caffeine pertain to quaternary carbon atoms; accordingly, their multiplicity is s (no hydrogen atoms are attached to the carbon). Their associated ^13^C NMR peaks will therefore be weak, which will add to the difficulty in correctly predicting substructures.

#### Broader Probabilistic Accuracy Assessments for This ML Model toward a General Case

Each type of molecular substructure-encoded representation that is defined by the neighbor approach has an accuracy that is influenced by the frequency of that substructure being present in the ^13^C NMR data set used to train our ML models. The more frequent a representation is, the greater the probability that a predicted molecular substructure that employs this representation matches the true substructure. Given that our aforementioned (top 1) accuracy denotes the proportion of instances in which the model’s highest-probability prediction matches the true substructure, this performance metric is most helpful for classifying spectra of molecules that contain common atom types. Yet, molecules that possess rarer atom types would benefit from a broader probabilistic evaluation of top 1, top 3, top 5, and top 10 accuracies, whose metrics assess whether the correct substructure appears within the one, three, five, or ten most probable candidates being predicted. These accuracies are stated for each type of molecular substructure representation that exists in the ^13^C NMR spectroscopy data set in the Supporting Information, SI. 5, using results from the CNN-based ML model that employs the neighbor approach.

Within this scope, consider the example of a molecular substructure labeled [7, 7, 17, 0, 1, 2, 1, 0], which follows the naming convention in Figure 2 and appears as row 7 of the Supporting Information, SI. 5. Its top 1 accuracy is only 28%; yet, its top 3 accuracy is far higher (87%). In a representative prediction, the ML model would rank the correct substructure (cf. row 7 of SI. 5) together with two others that have highly similar chemical motifs (denoted by rows 5 and 6 in SI. 5), among its three most probable outputs. This indicates that most errors stem from confusion within a statistically close family of related patterns rather than from random misclassification.

More generally, 112 types of molecular substructures occur more than 10 times in the ^13^C NMR spectroscopy data set. Given the above argument, we expect that molecules that are made up from such substructures will be characterized well by our CNN model using the neighbor approach, pending that its results are presented within a sufficiently broad envelope of probabilistic accuracy. We determined the appropriate level of reporting accuracy for such molecules by the following method. First, we defined a poorly characterized molecular substructure as the ML model correctly classifying it with a probability below 10%. The number of these 112 types of molecular substructures, which will be reported as being poorly characterized under this definition, declines sharply as the accuracy window widens: falling from 69 at the top 1 level to 35 at top 3, 20 at top 5, and 6 at top 10. These counts correspond to poor characterization rates of 62, 31, 18, and 5%, respectively. The substantial improvement seen from tracking top 1 to top 3 more generally corroborates our above notion that the ML model will often identify the correct molecular substructure within a small set of plausible alternatives, reflecting residual class overlap rather than a lack of chemical insight. At the same time, the consistently high performance of our CNN model beyond the top 3 accuracy level confirms that the substructure predictions from ^13^C NMR spectra capture meaningful and verifiable structural information even when the correct label is not ranked first.

Taken together, these observations support the overall validity and practical usefulness of our CNN model that uses the neighbor approach, especially in applications where a shortlist of candidate motifs can be examined or filtered in a downstream operation.

## Conclusions

This study has demonstrated the potential of neural networks to automate the characterization of the molecular substructure from ^13^C and ^1^H NMR spectral data. We have employed a vast set of experimental NMR spectra, present in the open database nmrshiftdb2, which has metadata on the experimental conditions: NMR field strength, temperature, and the sample’s solvent. Three types of neural-network models were considered based on MLP + RNN, MLP + LSTM, and CNN system architectures. For each neural-network architecture, we explored the adoption of two types of molecular substructure representations, using the neighbor and functional group approaches. The performance of each model was assessed using NMR spectral data that either do or do not incorporate metadata about key experimental conditions. A total of 20 neural-network architectures were assessed over this entire set of NMR probes, experimental conditions, substructure classification approaches, and types of neural-network architectures.

The MLP + LSTM model afforded the best statistical performance, achieving accuracy values that reached up to 88% for classifying ^13^C NMR data, using the neighbor approach, when it incorporated the three aforementioned experimental conditions. The inclusion of these metadata was important as can be seen by comparing these results with those of the analogous MLP + LSTM model, when trained on the same number of data but without experimental conditions, which reached an accuracy of 77%.

Notwithstanding these results, the CNN models yielded performance accuracies that were only slightly lower than those of the MLP + LSTM models, with the best-performing CNN model yielding 86% accuracy. Moreover, the CNN models achieved such performance levels while requiring only one-third of the computational run time compared to the MLP + LSTM models. Thus, CNN models offer greater practicality in being far more computationally efficient than MLP + LSTM models, at the cost of only a slight reduction in performance accuracy.

Model performance on ^1^H NMR spectra followed the same general trends as the findings on ^13^C NMR data. One distinction was that model performance on ^1^H NMR spectra was consistently lower than that on ^13^C NMR spectra. We expect this difference to arise because the nmrshiftdb2 database does not provide peak intensities for ^1^H NMR spectra; indeed, our ML models for ^1^H NMR spectra performed comparably to our ML models for ^13^C NMR spectra when they had been trained without their coupling-constant classification data. Overall, these results underscore the importance of including NMR field strength, temperature, and sample solvent metadata in NMR spectral analysis. Regarding the two types of molecular substructure representations, our novel neighbor approach gave marginally more accurate predictions from ^13^C NMR spectra than the functional group approach but the reverse was found for ^1^H NMR spectra.

Since this work has shown that deterministic neural-network models can be effective in automatically characterizing molecular substructures from NMR spectra, one could consider future work that explores the alternative use of more complex ML architectures, such as transformers. However, considerably more data would be needed for such a study. This issue is heightened by the fact that the best-performing models in this study arose from constraining their data sources to only 11 and 5% of the total ^13^C and ^1^H NMR spectral data that feature in the nmrshiftdb2 data set, respectively, so that we could incorporate metadata on all three key experimental conditions into our models.

The use of transformers holds the potential to bridge the molecular substructure identification capabilities of this work to that of elucidating an entire molecular structure. Nonetheless, such horizons need to be tempered by the computational cost of both the training and utility of a resulting automated analytical tool for characterizing NMR spectra. Meanwhile, the CNN models delivered by this work offer a new capability in automated NMR analysis that is important given the rise in autonomous robotic laboratories for chemistry where materials characterization is a crucial step in their materials discovery supply chain.

## Supplementary Material





## Data Availability

We have made available the source code and ML models for this work at https://github.com/Shiyun-Shelly-Liu/MPhil.

## Notes

Neighbor approach substructure label and details for ^13^C and ^1^H NMR spectra for all compounds; hyperparameter optimization tests and results for ^13^C and ^1^H complete and clean data sets, two classification methods, and different model architectures; model training and validation history plots for both spectra, complete and clean data sets, two classification methods, and different model architectures; model demonstration on selected four compounds (PDF)
Model performance for different neighbor approach substructures (XLS)
