# Supplementary material for: Automated Determination of the Molecular Substructure from Nuclear Magnetic Resonance Spectra Using Neural Networks
Source: J Chem Inf Model. 2025 Aug 13;65(16):8435–47. doi: 10.1021/acs.jcim.5c00499 (PMC12381849; doi:10.1021/acs.jcim.5c00499)
Supplement: Supplementary file 1 [file ci5c00499_si_001.pdf]

Supporting Information

Automated Determination of Molecular Substructure from NMR Spectra using Neural Networks

Shiyun Liu[1] and Jacqueline M. Cole[1,2,3*]

[1] *Cavendish Laboratory, Department of Physics, University of Cambridge, J. J. Thomson Avenue, Cambridge, CB3 0HE, U.K*

[2] *Science and Technology Facilities Council, Harwell Science and Innovation Campus, Didcot, Oxfordshire, OX14 0FA. U.K.*

[*] Address correspondence to: jmc61@cam.ac.uk

SI. 1 - Data Preparation

The integer labels of the neighbour approach for ^{13}C NMR spectral data are summarised in Table S1.1.

Table S1.1: Neighbour Approach Reference for ^{13}C NMR Spectral Data

**Substructure Label**	**Details**	**Substructure Label**	**Details**
0	0, 0, 0, 0, 0, 0, 0, 0	68	7, 32, 0, 0, 1, 2, 0, 0
1	7, 0, 0, 0, 1, 0, 0, 0	69	7, 32, 0, 0, 2, 1, 0, 0
2	7, 0, 0, 0, 2, 0, 0, 0	70	7, 32, 0, 0, 3, 1, 0, 0
3	7, 0, 0, 0, 3, 0, 0, 0	71	7, 32, 32, 0, 1, 1, 1, 0
4	7, 7, 0, 0, 1, 1, 0, 0	72	7, 32, 32, 0, 1, 1, 2, 0
5	7, 7, 0, 0, 1, 2, 0, 0	73	7, 32, 32, 0, 2, 1, 1, 0
6	7, 7, 0, 0, 1, 3, 0, 0	74	7, 32, 32, 32, 1, 1, 1, 1
7	7, 7, 0, 0, 2, 2, 0, 0	75	17, 0, 0, 0, 1, 0, 0, 0
8	7, 7, 7, 0, 1, 1, 1, 0	76	17, 0, 0, 0, 2, 0, 0, 0
9	7, 7, 7, 0, 1, 1, 2, 0	77	17, 0, 0, 0, 3, 0, 0, 0
10	7, 7, 7, 7, 1, 1, 1, 1	78	17, 17, 0, 0, 1, 1, 0, 0
11	7, 7, 7, 17, 1, 1, 1, 1	79	17, 17, 0, 0, 1, 2, 0, 0

Continued on next page

Table S1.1 – continued from previous page

**Substructure Label**	**Details**	**Substructure Label**	**Details**
12	7, 7, 7, 19, 1, 1, 1, 1	80	17, 17, 0, 0, 1, 3, 0, 0
13	7, 7, 7, 32, 1, 1, 1, 1	81	17, 17, 0, 0, 2, 2, 0, 0
14	7, 7, 17, 0, 1, 1, 1, 0	82	17, 17, 17, 0, 1, 1, 1, 0
15	7, 7, 17, 0, 1, 1, 2, 0	83	17, 17, 17, 0, 1, 1, 2, 0
16	7, 7, 17, 0, 1, 2, 1, 0	84	17, 17, 17, 17, 1, 1, 1, 1
17	7, 7, 17, 17, 1, 1, 1, 1	85	17, 17, 17, 19, 1, 1, 1, 1
18	7, 7, 17, 19, 1, 1, 1, 1	86	17, 17, 19, 0, 1, 1, 1, 0
19	7, 7, 17, 32, 1, 1, 1, 1	87	17, 17, 19, 0, 1, 1, 2, 0
20	7, 7, 19, 0, 1, 1, 1, 0	88	17, 17, 19, 0, 1, 2, 1, 0
21	7, 7, 19, 0, 1, 1, 2, 0	89	17, 17, 19, 19, 1, 1, 1, 1
22	7, 7, 19, 0, 1, 2, 1, 0	90	17, 17, 32, 0, 1, 1, 1, 0
23	7, 7, 19, 19, 1, 1, 1, 1	91	17, 17, 32, 0, 1, 1, 2, 0
24	7, 7, 19, 32, 1, 1, 1, 1	92	17, 17, 32, 0, 1, 2, 1, 0
25	7, 7, 32, 0, 1, 1, 1, 0	93	17, 19, 0, 0, 1, 1, 0, 0
26	7, 7, 32, 0, 1, 1, 2, 0	94	17, 19, 0, 0, 1, 2, 0, 0
27	7, 7, 32, 0, 1, 2, 1, 0	95	17, 19, 0, 0, 2, 1, 0, 0
28	7, 7, 32, 32, 1, 1, 1, 1	96	17, 19, 0, 0, 2, 2, 0, 0
29	7, 17, 0, 0, 1, 1, 0, 0	97	17, 19, 0, 0, 3, 1, 0, 0
30	7, 17, 0, 0, 1, 2, 0, 0	98	17, 19, 19, 0, 1, 1, 1, 0
31	7, 17, 0, 0, 1, 3, 0, 0	99	17, 19, 19, 0, 1, 1, 2, 0
32	7, 17, 0, 0, 2, 1, 0, 0	100	17, 19, 19, 0, 2, 1, 1, 0
33	7, 17, 0, 0, 2, 2, 0, 0	101	17, 19, 19, 32, 1, 1, 1, 1
34	7, 17, 0, 0, 3, 1, 0, 0	102	17, 19, 32, 0, 1, 1, 1, 0
35	7, 17, 17, 0, 1, 1, 1, 0	103	17, 19, 32, 0, 1, 1, 2, 0
36	7, 17, 17, 0, 1, 1, 2, 0	104	17, 19, 32, 0, 1, 2, 1, 0
37	7, 17, 17, 0, 2, 1, 1, 0	105	17, 19, 32, 0, 2, 1, 1, 0
38	7, 17, 17, 17, 1, 1, 1, 1	106	17, 32, 0, 0, 1, 1, 0, 0
39	7, 17, 17, 19, 1, 1, 1, 1	107	17, 32, 0, 0, 1, 2, 0, 0
40	7, 17, 17, 32, 1, 1, 1, 1	108	17, 32, 0, 0, 2, 1, 0, 0
41	7, 17, 19, 0, 1, 1, 1, 0	109	17, 32, 0, 0, 2, 2, 0, 0
42	7, 17, 19, 0, 1, 1, 2, 0	110	17, 32, 0, 0, 3, 1, 0, 0
43	7, 17, 19, 0, 1, 2, 1, 0	111	17, 32, 32, 0, 1, 1, 1, 0
44	7, 17, 19, 0, 2, 1, 1, 0	112	17, 32, 32, 0, 1, 1, 2, 0
45	7, 17, 19, 19, 1, 1, 1, 1	113	17, 32, 32, 0, 2, 1, 1, 0
46	7, 17, 19, 32, 1, 1, 1, 1	114	19, 0, 0, 0, 1, 0, 0, 0
47	7, 17, 32, 0, 1, 1, 1, 0	115	19, 19, 0, 0, 1, 1, 0, 0
48	7, 17, 32, 0, 1, 1, 2, 0	116	19, 19, 0, 0, 1, 2, 0, 0

Continued on next page

Table S1.1 – continued from previous page

**Substructure Label**	**Details**	**Substructure Label**	**Details**
49	7, 17, 32, 0, 1, 2, 1, 0	117	19, 19, 19, 0, 1, 1, 1, 0
50	7, 17, 32, 0, 2, 1, 1, 0	118	19, 19, 19, 0, 1, 1, 2, 0
51	7, 17, 32, 32, 1, 1, 1, 1	119	19, 19, 19, 19, 1, 1, 1, 1
52	7, 19, 0, 0, 1, 1, 0, 0	120	19, 19, 32, 0, 1, 1, 2, 0
53	7, 19, 0, 0, 1, 2, 0, 0	121	19, 19, 32, 0, 1, 2, 1, 0
54	7, 19, 0, 0, 2, 1, 0, 0	122	19, 19, 32, 32, 1, 1, 1, 1
55	7, 19, 0, 0, 2, 2, 0, 0	123	19, 32, 0, 0, 1, 1, 0, 0
56	7, 19, 0, 0, 3, 1, 0, 0	124	19, 32, 0, 0, 2, 1, 0, 0
57	7, 19, 19, 0, 1, 1, 1, 0	125	19, 32, 0, 0, 2, 2, 0, 0
58	7, 19, 19, 0, 1, 1, 2, 0	126	19, 32, 32, 0, 1, 1, 1, 0
59	7, 19, 19, 0, 2, 1, 1, 0	127	19, 32, 32, 0, 1, 1, 2, 0
60	7, 19, 19, 19, 1, 1, 1, 1	128	19, 32, 32, 0, 2, 1, 1, 0
61	7, 19, 19, 32, 1, 1, 1, 1	129	19, 32, 32, 32, 1, 1, 1, 1
62	7, 19, 32, 0, 1, 1, 1, 0	130	32, 0, 0, 0, 1, 0, 0, 0
63	7, 19, 32, 0, 1, 1, 2, 0	131	32, 0, 0, 0, 2, 0, 0, 0
64	7, 19, 32, 0, 1, 2, 1, 0	132	32, 32, 0, 0, 1, 1, 0, 0
65	7, 19, 32, 0, 2, 1, 1, 0	133	32, 32, 32, 0, 1, 1, 1, 0
66	7, 19, 32, 32, 1, 1, 1, 1	134	32, 32, 32, 0, 1, 1, 2, 0
67	7, 32, 0, 0, 1, 1, 0, 0	135	32, 32, 32, 32, 1, 1, 1, 1

For the neighbour approach using ^{1}H spectral data, there is a different atom-number labelling as shown in Table S1.2.

Table S1.2: Atom types and their corresponding numerical labels for ^{1}H spectral data. Note that the numerical label 24 was utilised to replace the individual labels for atom types other than C, N, or O, aimed at reducing the number of classes for certain models.

Atom Type	Numerical Label
Al	1
As	2
B	3
Br	4
C	5
Cl	6
F	7
Ga	8
Ge	9
H	10
Hg	11
I	12
Li	13
Mg	14
N	15
O	16
P	17
S	18
Se	19
Si	20
Sn	21
Te	22
Ti	23
Except for C, N, O	24

SI. 2 - Hyperparameter Optimisation Tests

The accuracy used in the grid search for hyperparameter fine-tuning was the validation accuracy. For the cross-validation, the training data was split into three folds. When the model was trained on one fold, the other two folds became the validation dataset. The optimisation for the learning rate and the momentum term beta_1 was done on most models. However, the default values were used to produce final results for their smoothness in the plots of train and validation histories.

Table S2.1: Fine-tuning tests of the batch size and number of epochs for the MLP+LSTM model with the complete ^{13}C dataset using the neighbour approach.

**Batch Size**	**Epoch**	**Accuracy**	**Standard Deviation**
3000	100	0.813292	0.003760
2000	100	0.812995	0.005325
4000	100	0.811030	0.003027
1500	100	0.809900	0.003523
3500	100	0.809102	0.002469
1600	100	0.808525	0.004603
6000	100	0.807564	0.004260
1200	100	0.807015	0.004448
1000	100	0.804103	0.002272
1300	100	0.803398	0.005223
300	100	0.802161	0.005594
300	50	0.801719	0.001145
3000	50	0.799556	0.002743
300	25	0.798641	0.000961
500	100	0.798513	0.004190
30	100	0.795480	0.005107
30	50	0.793504	0.004598
30	25	0.790115	0.000439
3000	25	0.789292	0.001085
300	5	0.782272	0.003043
30	5	0.775798	0.000733
3000	5	0.772706	0.003963
300	1	0.758880	0.001324
30	1	0.753108	0.006151
3000	1	0.561287	0.016319

Table S2.2: Fine-tuning tests of the optimisers for the MLP+LSTM model with the complete ^{13}C dataset using the neighbour approach.

**Optimiser**	**Accuracy**	**Standard Deviation**
Adam	0.810912	0.003512
Adamax	0.801743	0.001256
Nadam	0.790260	0.001003
RMSprop	0.782214	0.006827
Adagrad	0.694847	0.002395
SGD	0.629870	0.031645
Adadelta	0.433966	0.002947

Table S2.3: Fine-tuning tests of the learning rate and beta_1 for the MLP+LSTM model with the complete ^{13}C dataset using the neighbour approach.

**Learning Rate**	**Beta_1**	**Accuracy**	**Standard Deviation**
0.07	0.8	0.816087	0.001044
0.1	0.4	0.814374	0.003070
0.07	0.2	0.813752	0.000521
0.1	0.9	0.812636	0.004180
0.09	0.2	0.812563	0.002249
0.1	0.8	0.812107	0.004099
0.09	0.9	0.811997	0.001001
0.1	0	0.811897	0.000658
0.2	0.2	0.811727	0.001503
0.07	0.9	0.811320	0.001900
0.1	0.2	0.811292	0.002287
0.2	0.8	0.810266	0.002037
0.07	0.6	0.810200	0.003482
0.2	0.6	0.810066	0.001810
0.2	0.9	0.809624	0.002667
0.2	0.4	0.809624	0.002168
0.09	0.8	0.809841	0.001054
0.07	0	0.809800	0.001782
0.09	0.4	0.809451	0.002676
0.2	0	0.809271	0.001895
0.09	0.6	0.808819	0.003963
0.1	0.6	0.808566	0.004813
0.09	0	0.808546	0.001808
0.07	0.4	0.808414	0.003756

Table S2.4: Fine-tuning tests of the batch size for the MLP+LSTM model with the clean set of ^{13}C data using the neighbour approach.

**Batch Size**	**Accuracy**	**Standard Deviation**
600	0.873743	0.000594
1000	0.870452	0.004152
400	0.870452	0.005444
900	0.869657	0.002710
700	0.867667	0.002330
500	0.866365	0.007187
1500	0.865425	0.001369
2000	0.863942	0.000852
2500	0.863617	0.007580
100	0.861700	0.007477
3000	0.858915	0.008396
200	0.858662	0.015944
50	0.857324	0.007865
3500	0.856890	0.004750
800	0.853382	0.017986
4000	0.840073	0.019186

Table S2.5: Fine-tuning tests of the optimisers for the MLP+LSTM model with the clean set of ^{13}C data using the neighbour approach.

**Optimiser**	**Accuracy**	**Standard Deviation**
Adam	0.810912	0.003512
Adamax	0.801743	0.001256
Nadam	0.790260	0.001003
RMSprop	0.782214	0.006827
Adagrad	0.694847	0.002395
SGD	0.629870	0.031645
Adadelta	0.433966	0.002947

Table S2.6: Fine-tuning tests of the learning rate and beta_1 for the MLP+LSTM model with the clean set of ^{13}C data using the neighbour approach.

**Learning Rate**	**Beta_1**	**Accuracy**	**Standard Deviation**
0.09	0.7	0.866203	0.004711
0.2	0	0.864684	0.004503
0.01	0.2	0.863779	0.003263
0.001	0.6	0.863237	0.003528
0.01	0.9	0.863092	0.003895
0.2	0.9	0.862405	0.000714
0.001	0	0.861356	0.002459
0.2	0.6	0.861175	0.001359
0.001	0.2	0.860199	0.005030
0.01	0.6	0.859620	0.002443
0.2	0.2	0.859512	0.006296
0.01	0	0.858608	0.004521

Table S2.7: Fine-tuning tests of the batch size for the MLP+LSTM model with the set of ^{13}C data without any experimental condition specified using the neighbour approach.

**Batch Size**	**Accuracy**	**Standard Deviation**
2000	0.776406	0.006603
1000	0.769553	0.004123
500	0.768195	0.003946
4000	0.766717	0.007139
100	0.764591	0.005440
3000	0.763823	0.016174

Table S2.8: Fine-tuning tests of the optimisers for the MLP+LSTM model with the set of ^{13}C data without any experimental condition specified using the neighbour approach.

**Optimiser**	**Accuracy**	**Standard Deviation**
Adam	0.773393	0.003781
Nadam	0.773157	0.006782
Adamax	0.764473	0.008473
RMSprop	0.573718	0.096127

Table S2.9: Fine-tuning tests of the learning rate and beta_1 for the MLP+LSTM model with the set of ^{13}C data without any experimental condition specified using the neighbour approach.

**Learning Rate**	**Beta_1**	**Accuracy**	**Standard Deviation**
0.01	0.2	0.776465	0.004452
0.3	0.6	0.775874	0.009754
0.001	0.9	0.775283	0.004392
0.01	0.6	0.774928	0.009350
0.2	0.6	0.774752	0.006521
0.001	0	0.774220	0.007171
0.3	0.4	0.774161	0.005574
0.3	0.2	0.774574	0.007124
0.01	0	0.773629	0.006695
0.3	0.8	0.773629	0.001914
0.2	0.4	0.773275	0.005708
0.001	0.8	0.772329	0.008263
0.01	0.4	0.772802	0.005039
0.01	0.9	0.772270	0.004319
0.01	0.9	0.771976	0.001871
0.001	0.6	0.770911	0.008664
0.2	0.8	0.769375	0.009244
0.01	0.8	0.769140	0.003137
0.2	0.2	0.767368	0.002587
0.001	0.4	0.767309	0.005159
0.2	0	0.767072	0.009888
0.001	0.2	0.766187	0.008919
0.3	0.9	0.720171	0.070558

Table S2.10: Fine-tuning tests of the batch size and number of epochs for the CNN model with the complete ^{13}C dataset using the neighbour approach.

**Batch Size**	**Accuracy**	**Standard Deviation**
500	0.867306	0.000898
100	0.865570	0.003714
1000	0.865497	0.001545
2000	0.862206	0.000861
4000	0.851356	0.001771
3000	0.832442	0.017765

Table S2.11: Fine-tuning tests of the optimisers for the CNN model with the complete ^{13}C dataset using the neighbour approach.

**Optimiser**	**Accuracy**	**Standard Deviation**
Adamax	0.840760	0.014445
Adam	0.835950	0.022091
Nadam	0.833815	0.003286
RMSprop	0.815914	0.018775
SGD	0.806186	0.025516
Adagrad	0.572766	0.011422
Adadelta	0.396058	0.012404

Table S2.12: Fine-tuning tests of the learning rate and beta_1 for the CNN model with the complete ^{13}C dataset using the neighbour approach.

**Learning Rate**	**Beta_1**	**Accuracy**	**Standard Deviation**
0.4	0.01	0.802842	0.000790
0.2	0.2	0.802590	0.000674
0.6	0.01	0.801947	0.000998
0.6	0.001	0.801681	0.000582
0.8	0.01	0.801643	0.001219
0.6	0.2	0.801595	0.001452
0.2	0.001	0.801173	0.002981
0.4	0.001	0.801018	0.001097
0.8	0.001	0.800914	0.002407
0.2	0.01	0.800645	0.002968
0.8	0.05	0.800503	0.000748
0.4	0.05	0.800175	0.001282
0.2	0.05	0.800064	0.001010
0.6	0.05	0.799429	0.001037
0.4	0.2	0.798371	0.002550
0.8	0.2	0.797992	0.001428

Table S2.13: Fine-tuning tests of the batch size for the CNN model with the clean set of ^{13}C data using the neighbour approach.

**Batch Size**	**Accuracy**	**Standard Deviation**
100	0.863581	0.000942
1000	0.862315	0.000764
500	0.861772	0.004385
2000	0.858843	0.002520
4000	0.845787	0.006108
3000	0.802280	0.068042

Table S2.14: Fine-tuning tests of the optimisers for the CNN model with the clean set of ^{13}C data using the neighbour approach.

Optimiser	Accuracy	Standard Deviation
Adam	0.846836	0.005239
Adamax	0.839131	0.023472
Nadam	0.836420	0.000224
RMSprop	0.823111	0.013642
SGD	0.739533	0.142861
Adagrad	0.565931	0.005344
Adadelta	0.395950	0.027041

Table S2.15: Fine-tuning tests of the learning rate and beta_1 for the CNN model with the clean set of ^{13}C data using the neighbour approach.

Learning Rate	Beta_1	Accuracy	Standard Deviation
0.4	0.2	0.845714	0.007904
0.2	0.01	0.845714	0.001545
0.4	0.05	0.844810	0.001565
0.4	0.01	0.844593	0.003431
1	0.01	0.844196	0.007566
0.2	0.001	0.843002	0.001216
0.6	0.2	0.842134	0.004446
0.2	0.2	0.840687	0.001830
0.1	0.01	0.840036	0.009653
0.8	0.05	0.839204	0.007160
0.6	0.05	0.838481	0.005220
0.1	0.3	0.838300	0.003944
0.8	0.001	0.838083	0.007900
0.1	0.001	0.837613	0.001836
0.6	0.001	0.837035	0.005620
1	0.05	0.837035	0.005620
0.6	0.01	0.835914	0.008369
0.8	0.01	0.835588	0.020763
0.2	0.3	0.834901	0.006404
0.6	0.3	0.834828	0.009045
0.1	0.05	0.834611	0.007838
0.4	0.3	0.833635	0.003077
0.8	0.3	0.831609	0.015468
1	0.2	0.830416	0.001648
0.8	0.2	0.829765	0.013528
0.1	0.2	0.757110	0.118412
0.1	0.2	0.757110	0.118412

Table S2.16: Fine-tuning tests of the batch size for the CNN model with the set of ^{13}C data without any experimental condition specified using the neighbour approach.

**Batch Size**	**Accuracy**	**Standard Deviation**
1000	0.756085	0.009732
500	0.754313	0.013738
2000	0.749232	0.004672
100	0.720641	0.024193
3000	0.704397	0.020143
4000	0.601252	0.005202

Table S2.17: Fine-tuning tests of the optimisers for the CNN model with the set of ^{13}C data without any experimental condition specified using the neighbour approach.

**Optimiser**	**Accuracy**	**Standard Deviation**
Adamax	0.738067	0.004523
Adam	0.737654	0.007623
SGD	0.736058	0.004726
Nadam	0.735291	0.011196
RMSprop	0.718927	0.011585
Adagrad	0.671373	0.028014
Adadelta	0.466564	0.010571

Table S2.18: Fine-tuning tests of the learning rate and beta_1 for the CNN model with the set of ^{13}C data without any experimental condition specified using the neighbour approach.

**Learning Rate**	**Beta_1**	**Accuracy**	**Standard Deviation**
0.8	0.01	0.758566	0.008833
0.2	0.01	0.758034	0.007280
0.6	0.05	0.756498	0.004451
0.8	0.2	0.756440	0.017306
0.4	0.2	0.754076	0.007353
0.2	0.05	0.752658	0.006857
0.6	0.2	0.752600	0.008532
0.6	0.001	0.752186	0.007369
0.8	0.05	0.751595	0.003613
0.6	0.01	0.751418	0.002975
0.4	0.001	0.748877	0.014727
0.2	0.001	0.748168	0.007788
0.8	0.001	0.746692	0.011610
0.4	0.05	0.745215	0.018291
0.2	0.2	0.744034	0.006011
0.4	0.01	0.740195	0.019326

Table S2.19: Fine-tuning tests of the batch size and number of epochs for the MLP+RNN model with the complete ^{13}C dataset using the neighbour approach.

**Batch Size**	**Accuracy**	**Standard Deviation**
4000	0.795953	0.004240
3000	0.795611	0.002565
2000	0.789127	0.005188
100	0.781284	0.009164
500	0.776607	0.008645
1000	0.775169	0.008298

Table S2.20: Fine-tuning tests of the optimisers for the MLP+RNN model with the complete ^{13}C dataset using the neighbour approach.

**Optimiser**	**Accuracy**	**Standard Deviation**
Adamax	0.802973	0.003891
Adam	0.798213	0.006690
Nadam	0.792288	0.007212
RMSprop	0.772896	0.004530
Adagrad	0.672246	0.021020
SGD	0.650526	0.029757
Adadelta	0.446300	0.003543

Table S2.21: Fine-tuning tests of the learning rate and beta_1 for the MLP+RNN model with the complete ^{13}C dataset using the neighbour approach.

**Learning Rate**	**Beta_1**	**Accuracy**	**Standard Deviation**
0.001	0.5	0.804231	0.002747
0.2	0.5	0.803066	0.001611
0.01	0.9	0.802103	0.005241
0.01	0.5	0.801819	0.002205
0.001	0.9	0.801273	0.000159
0.2	0.9	0.797677	0.005755

Table S2.22: Fine-tuning tests of the batch size for the MLP+RNN model with the clean set of ^{13}C data using the neighbour approach.

**Batch Size**	**Accuracy**	**Standard Deviation**
1000	0.861266	0.004525
3000	0.860434	0.008417
500	0.858083	0.011517
4000	0.849150	0.003663
100	0.846076	0.004596
2000	0.843871	0.019214

Table S2.23: Fine-tuning tests of the optimisers for the MLP+RNN model with the clean set of ^{13}C data using the neighbour approach.

Optimiser	Accuracy	Standard Deviation
Adamax	0.853996	0.005122
Nadam	0.852803	0.003900
Adam	0.850560	0.006936
RMSprop	0.769473	0.060772
SGD	0.503834	0.006015
Adagrad	0.481230	0.011468
Adadelta	0.303436	0.016937

Table S2.24: Fine-tuning tests of the learning rate and beta_1 for the MLP+RNN model with the clean set of ^{13}C data using the neighbour approach.

Learning Rate	Beta_1	Accuracy	Standard Deviation
0.01	0.8	0.869910	0.000927
0.3	0.2	0.868101	0.005415
0.01	0.2	0.866872	0.003547
0.2	0.8	0.866438	0.007488
0.01	0.6	0.866655	0.002191
0.2	0.6	0.865931	0.000871
0.3	0.8	0.863761	0.003224
0.3	0.6	0.863978	0.006299
0.001	0.2	0.863219	0.006685
0.2	0.6	0.865931	0.000871
0.001	0.6	0.864593	0.004616
0.2	0.2	0.861736	0.004552
0.001	0.8	0.626410	0.336685

Table S2.25: Fine-tuning tests of the batch size for the MLP+LSTM model with the complete ^{13}C dataset using the functional group approach.

Batch Size	Accuracy	Standard Deviation
1000	0.862122	0.005960
3000	0.857057	0.007365
2000	0.854753	0.002796
4000	0.847256	0.001580
100	0.844620	0.002111
500	0.840771	0.009110

Table S2.26: Fine-tuning tests of the optimisers for the MLP+LSTM model with the complete ^{13}C dataset using the functional group approach.

Optimiser	Accuracy	Standard Deviation
Nadam	0.859233	0.001484
Adam	0.852669	0.010271
Adamax	0.834767	0.003381
RMSprop	0.834259	0.008828

Table S2.27: Fine-tuning tests of the learning rate and beta_1 for the MLP+LSTM model with the complete ^{13}C dataset using the functional group approach.

Learning Rate	Beta_1	Accuracy	Standard Deviation
0.001	0.6	0.854863	0.004502
0.2	0.2	0.852393	0.000899
0.001	0	0.850711	0.001307
0.001	0.2	0.849591	0.001748
0.001	0.9	0.847874	0.003567
0.01	0.2	0.847778	0.002498
0.01	0.6	0.847584	0.001372
0.01	0	0.846396	0.002414
0.2	0.9	0.844813	0.002748
0.01	0.9	0.844672	0.004248
0.2	0.6	0.844585	0.002246
0.2	0	0.844140	0.001620

Table S2.28: Fine-tuning tests of the batch size for the CNN model with the complete ^{13}C dataset using the functional group approach.

Batch Size	Accuracy	Standard Deviation
2000	0.846758	0.000747
1000	0.842454	0.001337
100	0.842513	0.003833
500	0.839199	0.003045
3000	0.836691	0.004196
4000	0.835797	0.004250

Table S2.29: Fine-tuning tests of the optimisers for the CNN model with the complete ^{13}C dataset using the functional group approach.

Optimiser	Accuracy	Standard Deviation
Nadam	0.847847	0.000345
Adam	0.844520	0.002982
Adamax	0.841407	0.001769

Table S2.30: Fine-tuning tests of the learning rate and beta_1 for the CNN model with the complete ^{13}C dataset using the functional group approach.

**Learning Rate**	**Beta_1**	**Accuracy**	**Standard Deviation**
0.01	0.4	0.837939	0.003078
0.05	0.9	0.836239	0.004263
0.01	0.2	0.835805	0.003544
0.05	0.4	0.833635	0.001407
0.05	0.6	0.833490	0.003781
0.01	0.6	0.832839	0.002708
0.1	0.8	0.832550	0.004210
0.1	0.4	0.831682	0.007406
0.1	0.6	0.831140	0.006713
0.2	0.8	0.830777	0.002844
0.001	0.4	0.830126	0.004892
0.3	0.8	0.828933	0.004967
0.2	0.9	0.828680	0.005935
0.3	0.2	0.828463	0.004478
0.05	0.2	0.827957	0.008309
0.001	0.2	0.827776	0.003016
0.001	0.2	0.827414	0.002172
0.3	0.9	0.826763	0.004127
0.2	0.2	0.826257	0.007758
0.2	0.6	0.824702	0.003376
0.2	0.9	0.823074	0.000879
0.01	0.2	0.829910	0.002386

Table S2.31: Fine-tuning tests of the batch size for the MLP+LSTM model with the clean set of ^{13}C data using the functional group approach.

**Batch Size**	**Accuracy**	**Standard Deviation**
500	0.855334	0.005167
1000	0.854213	0.001298
2000	0.843798	0.007737
3000	0.839928	0.004081
100	0.827234	0.010842
4000	0.818590	0.027128

Table S2.32: Fine-tuning tests of the optimisers for the MLP+LSTM model with the clean set of ^{13}C data using the functional group approach.

**Optimiser**	**Accuracy**	**Standard Deviation**
Adam	0.854647	0.007133
Adamax	0.852477	0.005331
Nadam	0.847921	0.002676
RMSprop	0.836673	0.012396
SGD	0.761012	0.016880
Adagrad	0.750488	0.006538
Adadelta	0.729186	0.010296

Table S2.33: Fine-tuning tests of the learning rate and beta_1 for the MLP+LSTM model with the clean set of ^{13}C data using the functional group approach.

**Learning Rate**	**Beta_1**	**Accuracy**	**Standard Deviation**
0.001	0.9	0.858951	0.006777
0.001	0	0.856058	0.005462
0.2	0.6	0.855913	0.003307
0.01	0.6	0.855262	0.005896
0.2	0.2	0.854647	0.005809
0.001	0.2	0.853924	0.003992
0.01	0.9	0.853671	0.009973
0.01	0.2	0.853128	0.007360
0.2	0.6	0.853056	0.004995
0.2	0	0.853056	0.003671
0.01	0	0.850307	0.004026
0.2	0.9	0.849657	0.006113

Table S2.34: Fine-tuning tests of the batch size for the CNN model with the clean set of ^{13}C data using the functional group approach.

**Batch Size**	**Accuracy**	**Standard Deviation**
1000	0.843400	0.007933
2000	0.840868	0.003377
500	0.840759	0.010320
3000	0.839747	0.002684
100	0.831899	0.003977
4000	0.834575	0.006558

Table S2.35: Fine-tuning tests of the optimisers for the CNN model with the clean set of ^{13}C data using the functional group approach.

**Optimiser**	**Accuracy**	**Standard Deviation**
Adam	0.836383	0.003548
Adamax	0.834900	0.008667
Nadam	0.817975	0.018982

Table S2.36: Fine-tuning tests of the batch size and number of epochs for the MLP+LSTM model with the complete ^{1}H dataset using the neighbour approach.

**Batch Size**	**Accuracy**	**Standard Deviation**
2000	0.616354	0.004425
4000	0.616193	0.013374
3000	0.607058	0.007942
1000	0.597963	0.013491
500	0.593749	0.005741
100	0.577274	0.002028

Table S2.37: Fine-tuning tests of the optimisers for the MLP+LSTM model with the complete ^{1}H dataset using the neighbour approach.

**optimiser**	**Accuracy**	**Standard Deviation**
Adamax	0.636721	0.001241
RMSprop	0.632476	0.004233
Nadam	0.620911	0.004929
Adam	0.619681	0.004891
SGD	0.575912	0.003291
Adagrad	0.543194	0.002226
Adadelta	0.472010	0.010765

Table S2.38: Fine-tuning tests of the learning rate and beta_1 for the MLP+LSTM model with the complete ^{1}H dataset using the neighbour approach.

**Learning Rate**	**Beta_1**	**Accuracy**	**Standard Deviation**
0	0.001	0.631861	0.003984
0	0.2	0.627969	0.002375
0.4	0.01	0.627858	0.004870
0.9	0.01	0.626780	0.010714
0.8	0.01	0.626225	0.004844
0.9	0.3	0.625207	0.004680
0.8	0.001	0.624582	0.006321
0	0.3	0.624168	0.009932
0.2	0.2	0.623382	0.005214
0.4	0.001	0.622847	0.007237
0.6	0.01	0.622696	0.009021
0.2	0.001	0.622595	0.002293
0.6	0.001	0.620952	0.007704
0.6	0.2	0.620932	0.012148
0.2	0.3	0.620085	0.006680
0	0.01	0.619994	0.004038
0.9	0.2	0.619560	0.007060
0.9	0.001	0.618502	0.013925
0.8	0.3	0.618129	0.009141
0.6	0.3	0.615739	0.003262
0.4	0.2	0.613612	0.010017

Table S2.39: Fine-tuning tests of the batch size and number of epochs for the CNN model with the complete ^{1}H dataset using the neighbour approach.

**Batch Size**	**Accuracy**	**Standard Deviation**
500	0.623674	0.001497
1000	0.620629	0.002382
2000	0.615457	0.003908
3000	0.612886	0.003849
4000	0.615386	0.002001
100	0.600887	0.005779

Table S2.40: Fine-tuning tests of the optimisers for the CNN model with the complete ^{1}H dataset using the neighbour approach.

**optimiser**	**Accuracy**	**Standard Deviation**
Nadam	0.622303	0.003753
Adam	0.622222	0.003082
Adamax	0.619560	0.000863

Table S2.41: Fine-tuning tests of the learning rate and beta_1 for the CNN model with the complete ^{1}H dataset using the neighbour approach.

**Learning Rate**	**Beta_1**	**Accuracy**	**Standard Deviation**
0.01	0.8	0.630954	0.002624
0.2	0.2	0.630913	0.003516
0.05	0.6	0.630480	0.000959
0.2	0.8	0.629986	0.003144
0.05	0.4	0.629673	0.001319
0.01	0.4	0.629109	0.004977
0.001	0.4	0.629089	0.004977
0.01	0.2	0.628806	0.004944
0.001	0.2	0.628231	0.002252
0.05	0.8	0.627717	0.000488
0.01	0.6	0.627334	0.002765
0.2	0.2	0.627334	0.005695
0.2	0.4	0.626759	0.002746
0.001	0.6	0.626497	0.002139
0.05	0.2	0.628776	0.005568

Table S2.42: Fine-tuning tests of the batch size for the MLP+LSTM model with the clean set of ^{1}H data using the neighbour approach.

**Batch Size**	**Accuracy**	**Standard Deviation**
100	0.735250	0.005868
2000	0.717772	0.002427
1000	0.696656	0.027606
3000	0.694101	0.001156
500	0.686444	0.038499
4000	0.685000	0.012927

Table S2.43: Fine-tuning tests of the optimisers for the MLP+LSTM model with the clean set of ^{1}H data using the neighbour approach.

**optimiser**	**Accuracy**	**Standard Deviation**
Adam	0.735799	0.010528
Nadam	0.728698	0.005874
Adamax	0.717954	0.006527
RMSprop	0.686820	0.012676
SGD	0.634743	0.007062
Adagrad	0.597417	0.016174
Adadelta	0.472140	0.012351

Table S2.44: Fine-tuning tests of the learning rate and beta_1 for the MLP+LSTM model with the clean set of ^{1}H data using the neighbour approach.

**Learning Rate**	**Beta_1**	**Accuracy**	**Standard Deviation**
0.001	0.4	0.740350	0.003553
0.01	0.4	0.740167	0.004995
0.001	0	0.739986	0.004676
0.2	0.4	0.739439	0.004075
0.3	0.4	0.739257	0.003673
0.001	0.6	0.738893	0.007135
0.01	0.2	0.738530	0.007312
0.001	0.9	0.737984	0.004078
0.3	0.2	0.737437	0.004999
0.3	0.6	0.736160	0.010121
0.2	0.9	0.735435	0.010669
0.3	0.9	0.734524	0.006632
0.3	0.8	0.734157	0.005388
0.01	0.9	0.733795	0.008484
0.2	0.6	0.733795	0.002765
0.2	0.8	0.732340	0.009000
0.01	0.6	0.730518	0.003702
0.2	0.2	0.728517	0.014802
0.001	0.2	0.728517	0.012211
0.01	0.8	0.727969	0.005951
0.001	0.8	0.720865	0.005499

Table S2.45: Fine-tuning tests of the batch size for the CNN model with the clean set of ^{1}H data using the neighbour approach.

**Batch Size**	**Accuracy**	**Standard Deviation**
100	0.712309	0.009604
300	0.707029	0.005688
100	0.705937	0.011845
400	0.704844	0.003214
1000	0.704844	0.001212
500	0.703752	0.004302
200	0.703203	0.007302
2000	0.694648	0.009413
3000	0.690276	0.006633
4000	0.670977	0.005505

Table S2.46: Fine-tuning tests of the optimisers for the CNN model with the clean set of ^{1}H data using the neighbour approach.

optimiser	Accuracy	Standard Deviation
Nadam	0.704662	0.004481
Adam	0.702475	0.006149
Adamax	0.691916	0.002852

Table S2.47: Fine-tuning tests of the learning rate and beta_1 for the CNN model with the clean set of ^{1}H data using the neighbour approach.

Learning Rate	Beta_1	Accuracy	Standard Deviation
0.1	0.2	0.632441	0.005304
0.01	0.8	0.630954	0.002624
0.2	0.2	0.630913	0.003516
0.05	0.6	0.630480	0.000959
0.2	0.4	0.629946	0.003728
0.2	0.4	0.629673	0.001319
0.01	0.4	0.629109	0.004977
0.01	0.6	0.628806	0.004944
0.05	0.2	0.628776	0.005568
0.2	0.6	0.628443	0.005695
0.1	0.6	0.627717	0.000488
0.001	0.6	0.627334	0.002765
0.01	0.6	0.627334	0.002765
0.2	0.4	0.626759	0.002746
0.2	0.2	0.626497	0.002139
0.01	0.4	0.623089	0.008749
0.2	0.2	0.622303	0.003753
0.001	0.6	0.622222	0.003082
0.01	0.2	0.620629	0.002382
0.2	0.2	0.619560	0.000863
0.2	0.2	0.618567	0.006209
0.01	0.6	0.616578	0.006048
0.05	0.2	0.614789	0.003090
0.2	0.4	0.614173	0.008114
0.1	0.2	0.613955	0.005467
0.2	0.8	0.612374	0.005307
0.001	0.6	0.611813	0.004623

Table S2.48: Fine-tuning tests of the batch size and number of epochs for the MLP+LSTM model with the complete ^{1}H dataset using the functional group approach.

Batch Size	Accuracy	Standard Deviation
4000	0.735985	0.003055
3000	0.729401	0.005288
2000	0.728736	0.005889
1000	0.726618	0.003680
500	0.719722	0.010364
100	0.703831	0.002644

Table S2.49: Fine-tuning tests of the optimisers for the MLP+LSTM model with the complete ^{1}H dataset using the functional approach.

optimiser	Accuracy	Standard Deviation
Nadam	0.737024	0.003080
Adam	0.735824	0.002558
Adamax	0.730954	0.002706

Table S2.50: Fine-tuning tests of the batch size for the MLP+LSTM model with the clean set of ^{1}H data using the functional group approach.

Batch Size	Accuracy	Standard Deviation
100	0.786778	0.009265
1000	0.783867	0.004657
500	0.782591	0.007343
200	0.782957	0.009870
400	0.780586	0.015905
300	0.778216	0.026021
2000	0.774216	0.007968

Table S2.51: Fine-tuning tests of the optimisers for the MLP+LSTM model with the clean set of ^{1}H data using the functional group approach.

optimiser	Accuracy	Standard Deviation
Nadam	0.789875	0.006549
Adam	0.782409	0.008353
Adamax	0.774034	0.004634

Table S2.52: Fine-tuning tests of the batch size and number of epochs for the CNN model with the complete ^{1}H dataset using the functional group approach.

**Batch Size**	**Accuracy**	**Standard Deviation**
500	0.723714	0.002684
1000	0.720186	0.001720
100	0.715467	0.001382
2000	0.712321	0.002080
4000	0.709931	0.001732
3000	0.706282	0.005940

Table S2.53: Fine-tuning tests of the optimisers for the CNN model with the complete ^{1}H dataset using the functional group approach.

**optimiser**	**Accuracy**	**Standard Deviation**
Adam	0.722817	0.001138
Nadam	0.721032	0.002786
Adamax	0.713299	0.002964

Table S2.54: Fine-tuning tests of the batch size for the CNN model with the clean set of ^{1}H data using the functional group approach.

**Batch Size**	**Accuracy**	**Standard Deviation**
100	0.741989	0.003352
500	0.733614	0.006790
1000	0.723596	0.010862
2000	0.716314	0.002967
3000	0.706666	0.007380
4000	0.702114	0.007009

Table S2.55: Fine-tuning tests of the optimisers for the CNN model with the clean set of ^{1}H data using the functional group approach.

**optimiser**	**Accuracy**	**Standard Deviation**
Nadam	0.758193	0.005519
Adamax	0.743628	0.008490
Adam	0.739072	0.012975

Details of optimum hyperparameters used in the final ML models

Table S2.56: Hyperparameter optimisation results of the MLP+LSTM Model for the neighbour approach using ^{13}C NMR spectra with multiplicities as estimations for intensities.

Hyperparameters	Complete Data	Clean Data	Unspecified Data
**Batch Size**	3000	600	2000
**No. of Epochs**	100	50	100
**Optimiser**	Adam	Adam	Adam
**Learning Rate**	0.001	0.001	0.001
**Momentum**	0.9	0.9	0.9

Table S2.57: Hyperparameter optimisation results of the CNN model for the neighbour approach using ^{13}C NMR spectra with multiplicities as estimations for intensities.

Hyperparameters	Complete Data	Clean Data	Unspecified Data
**Batch Size**	500	100	1000
**No. of Epochs**	150	120	250
**Optimiser**	Adamax	Adam	Adamax
**Learning Rate**	0.001	0.001	0.001
**Momentum**	0.9	0.9	0.9

Table S2.58: Hyperparameter optimisation results of the MLP+RNN model for the neighbour approach using ^{13}C NMR spectra with multiplicities as estimations for intensities.

Hyperparameters	Complete Data	Clean Data
**Batch Size**	4000	1000
**No. of Epochs**	110	50
**Optimiser**	Adamax	Adamax
**Learning Rate**	0.001	0.001
**Momentum**	0.9	0.9

Table S2.59: Hyperparameter optimisation results of the MLP+LSTM model for the functional group approach using ^{13}C NMR spectra with multiplicities as estimations for intensities.

Hyperparameters	Complete Data	Clean Data
**Batch Size**	1000	500
**No. of Epochs**	190	100
**Optimiser**	Nadam	Adam
**Learning Rate**	0.001	0.001
**Momentum**	0.9	0.9

Table S2.60: Hyperparameter optimisation results of the CNN model for the functional group approach using ^{13}C NMR spectra with multiplicities as estimations for intensities.

**Hyperparameters**	**Complete Data**	**Clean Data**
**Batch Size**	2000	1000
**No. of Epochs**	250	350
**Optimiser**	Nadam	Adam
**Learning Rate**	0.001	0.001
**Momentum**	0.9	0.9

Table S2.61: Hyperparameter optimisation results of the MLP+LSTM model for the neighbour approach using ^{1}H NMR spectra.

**Hyperparameters**	**Complete Data**	**Clean Data**
**Batch Size**	2000	100
**No. of Epochs**	290	190
**Optimiser**	Adamax	Adam
**Learning Rate**	0.001	0.001
**Momentum**	0.9	0.9

Table S2.62: Hyperparameter optimisation results of the CNN model for the neighbour approach using ^{1}H NMR spectra.

**Hyperparameters**	**Complete Data**	**Clean Data**
**Batch Size**	500	100
**No. of Epochs**	200	250
**Optimiser**	Nadam	Nadam
**Learning Rate**	0.001	0.001
**Momentum**	0.9	0.9

Table S2.63: Hyperparameter optimisation results of the MLP+LSTM model for the functional group approach using ^{1}H NMR spectra.

**Hyperparameters**	**Complete Data**	**Clean Data**
**Batch Size**	4000	100
**No. of Epochs**	200	250
**Optimiser**	Nadam	Nadam
**Learning Rate**	0.001	0.001
**Momentum**	0.9	0.9

Table S2.64: Hyperparameter optimisation results of the CNN model for the functional group approach using ^{1}H NMR spectra.

**Hyperparameters**	**Complete Data**	**Clean Data**
**Batch Size**	500	100
**No. of Epochs**	100	250
**Optimiser**	Adam	Nadam
**Learning Rate**	0.001	0.001
**Momentum**	0.9	0.9

SI. 3 - Model Training and Validation History

Plots of model accuracy and model loss for the learning rate and momentum parameters for MLP+LSTM model using optimised learning rates and beta_1 (obatained by grid search and default values).

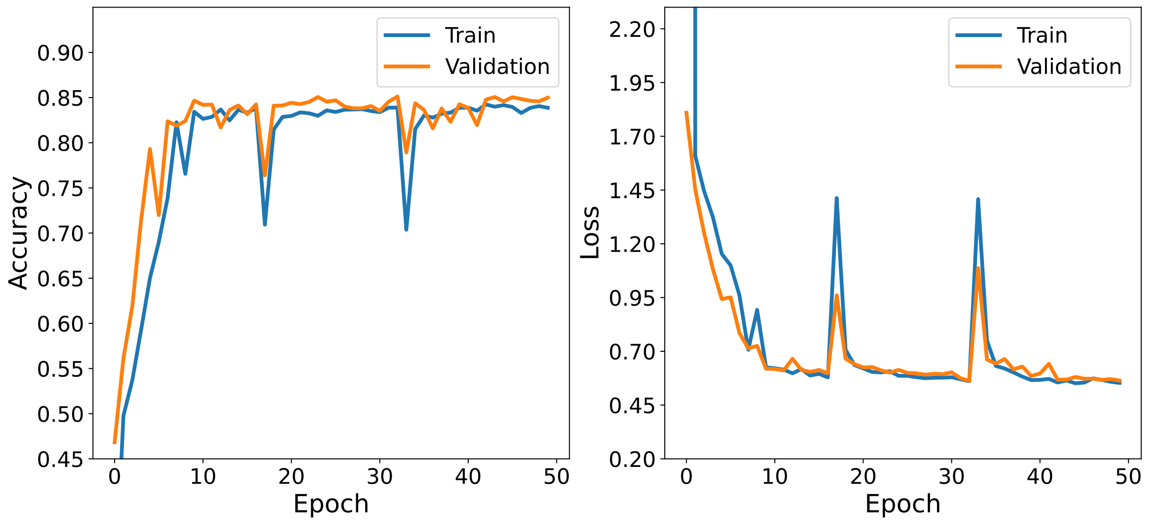

(a) The model history of accuracies (left) and losses (right) using the optimised learning rate (0.09) and beta_1 (0.7) from a grid search.

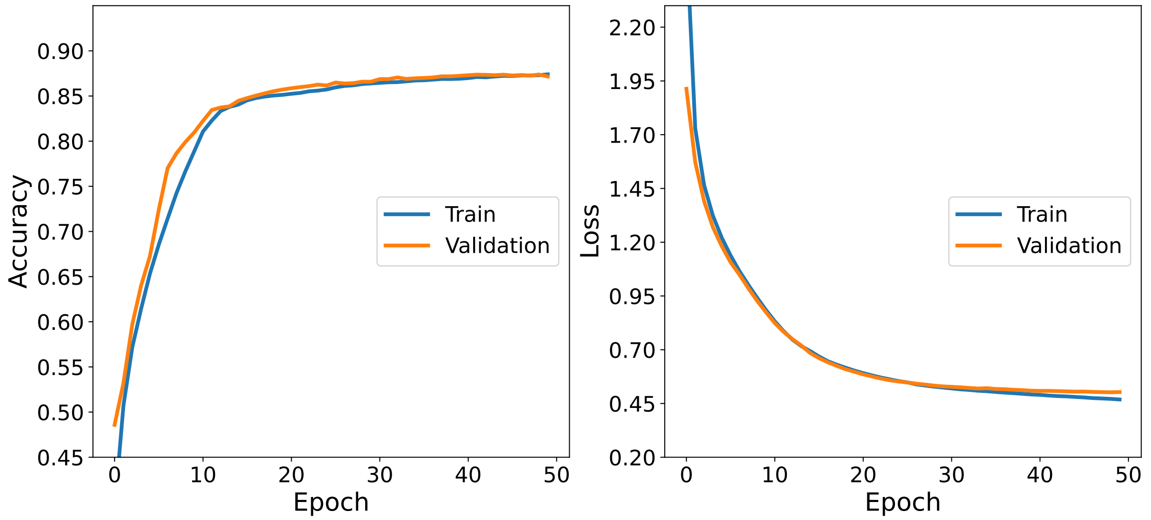

(b) The model history of accuracies (left) and losses (right) using default learning rate (0.001) and beta_1 (0.9).

Figure S3.1: A comparison of the model history of accuracies and losses for MLP+LSTM model using optimised learning rates and beta_1 from a grid search and that from default. ^{13}C spectral data with three experimental conditions specified was used. Both sets of parameters yielded an accuracy of 86%.

The training and validation history of the accuracy and loss for each model as a function of the number of the epochs are given here.

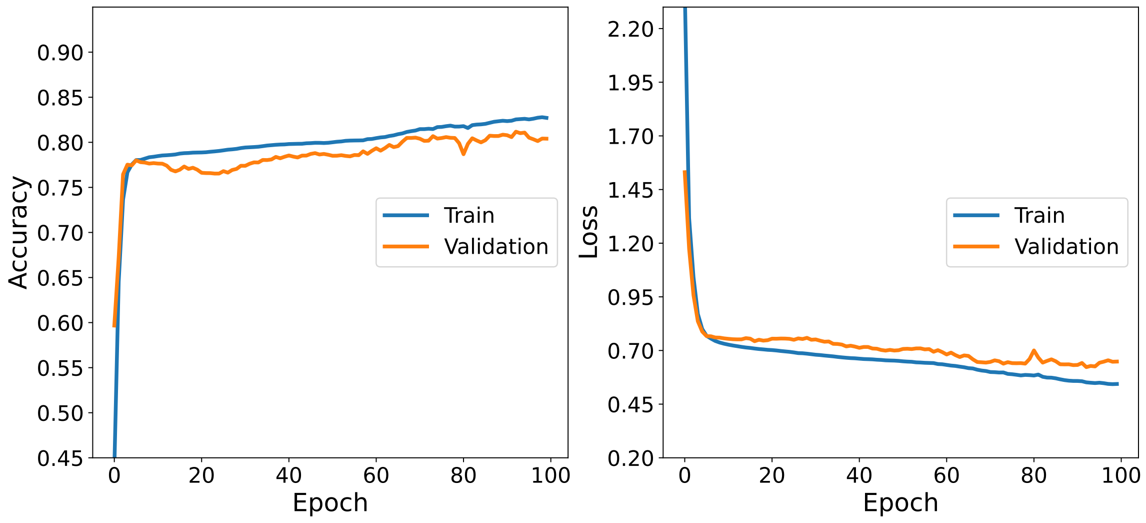

Figure S3.2: The training and validation history of the accuracy and loss for the MLP+LSTM model using the full set of ^{13}C spectral data with the neighbour approach.

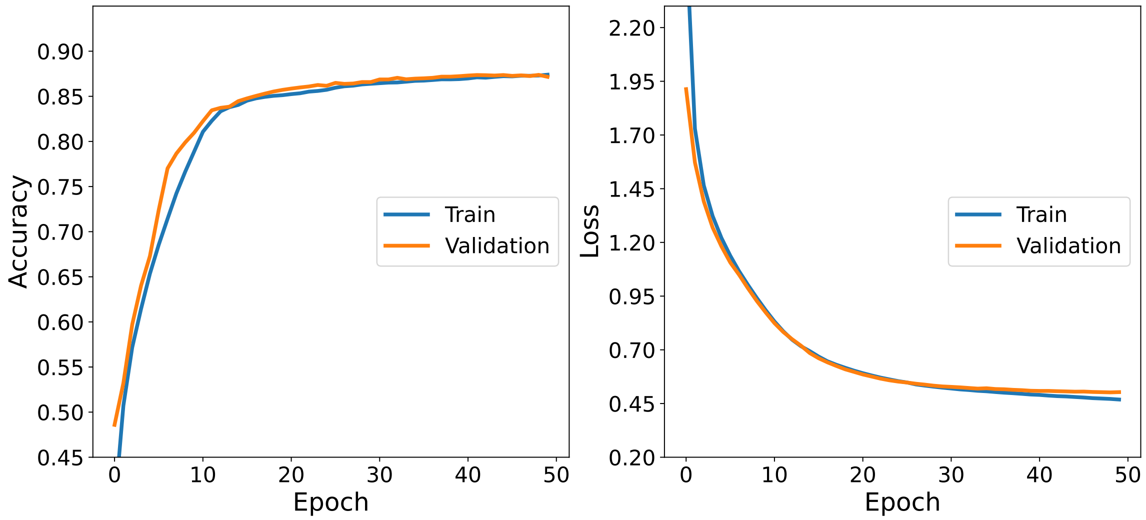

Figure S3.3: The training and validation history of the accuracy and loss for the MLP+LSTM model using the set of ^{13}C spectral data with three specified experimental conditions with the neighbour approach.

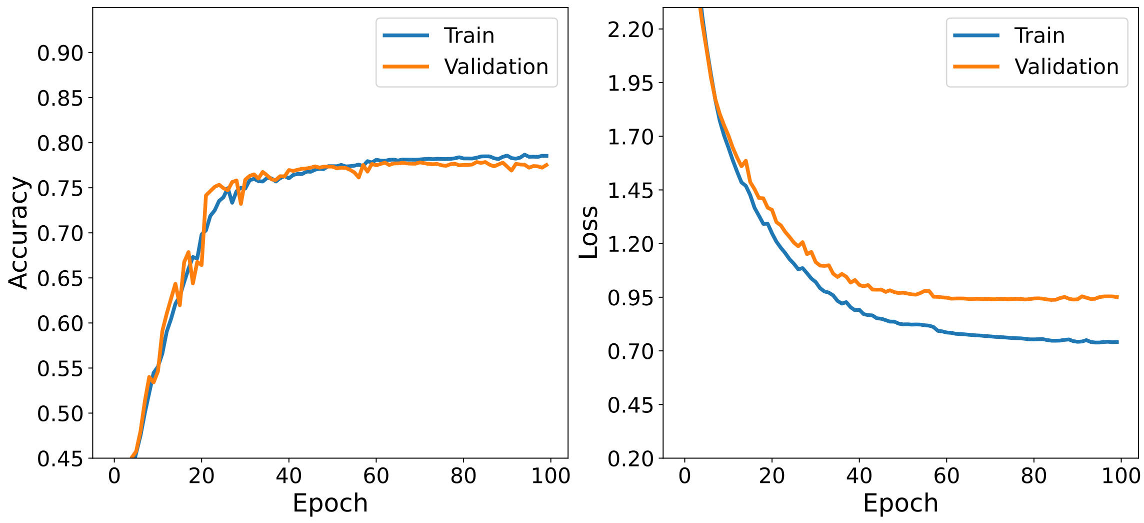

Figure S3.4: The training and validation history of the accuracy and loss for the MLP+LSTM model using the set of ^{13}C spectral data with three experimental conditions unspecified with the neighbour approach.

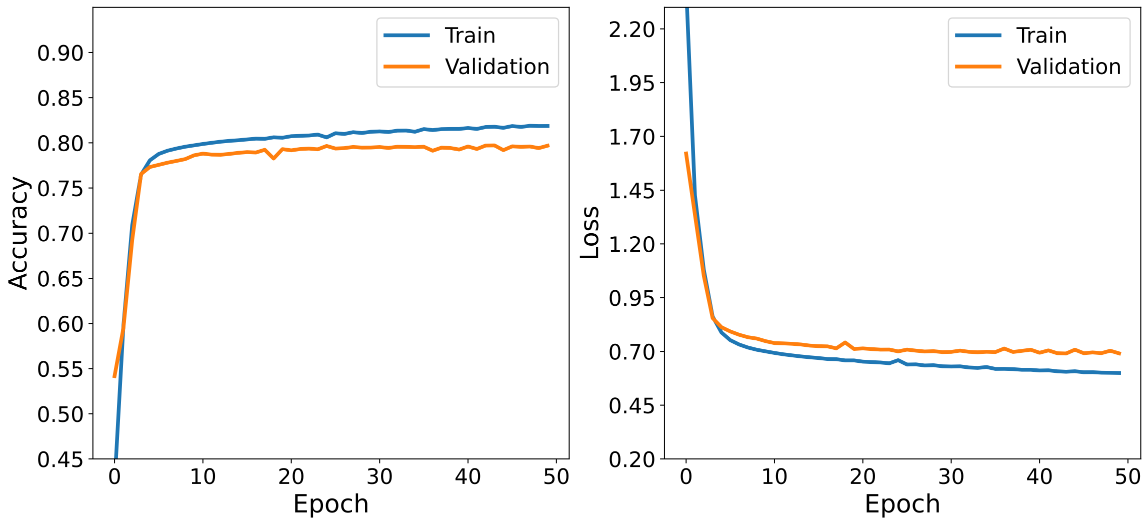

Figure S3.5: The training and validation history of the accuracy and loss for the CNN model using the full set of ^{13}C spectral data with the neighbour approach.

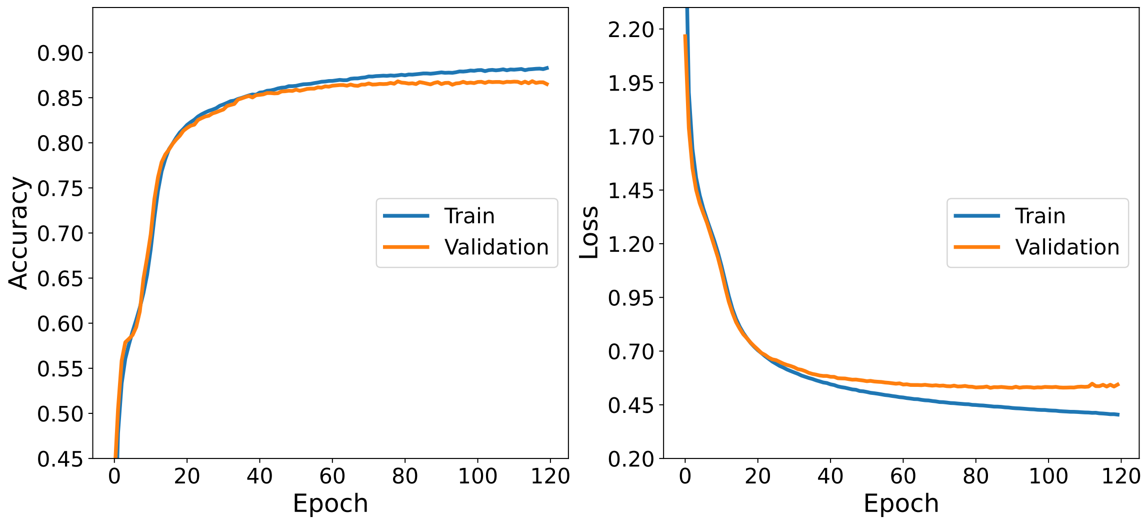

Figure S3.6: The training and validation history of the accuracy and loss for the CNN model using the set of ^{13}C spectral data with three specified experimental conditions with the neighbour approach.

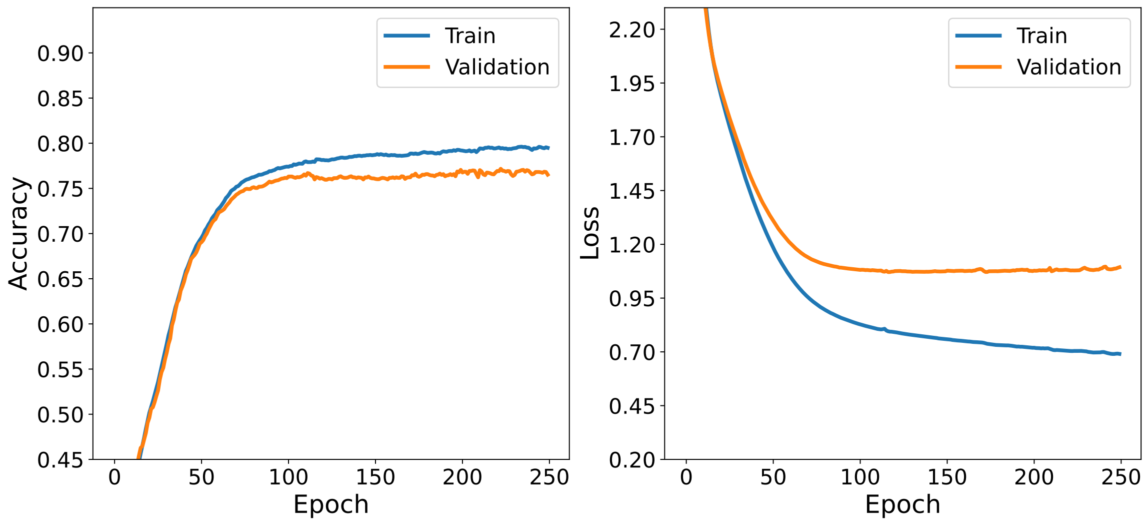

Figure S3.7: The training and validation history of the accuracy and loss for the CNN model using the set of ^{13}C spectral data with three experimental conditions unspecified with the neighbour approach.

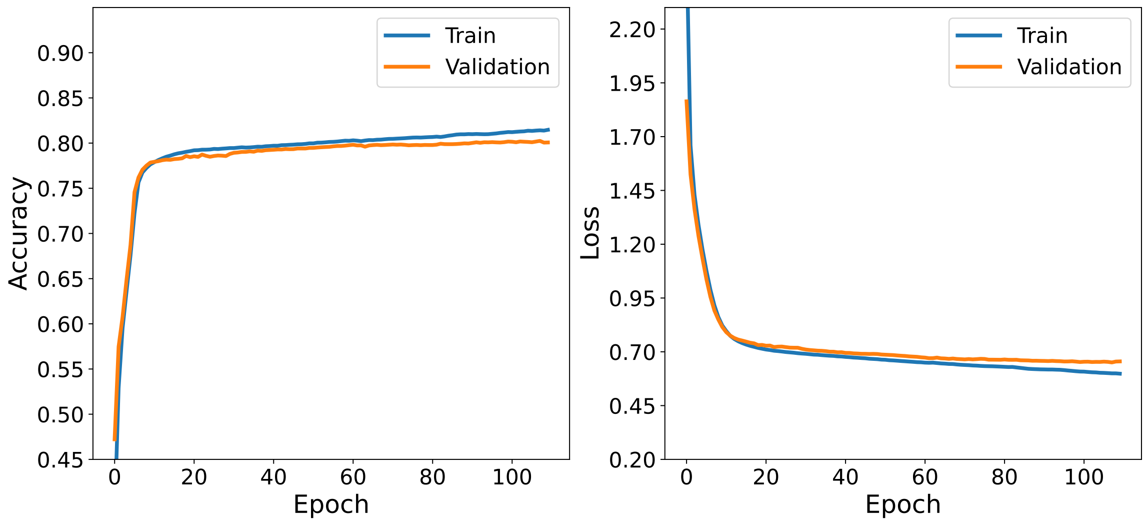

Figure S3.8: The training and validation history of the accuracy and loss for the MLP+RNN model using the full set of ^{13}C spectral data with the neighbour approach.

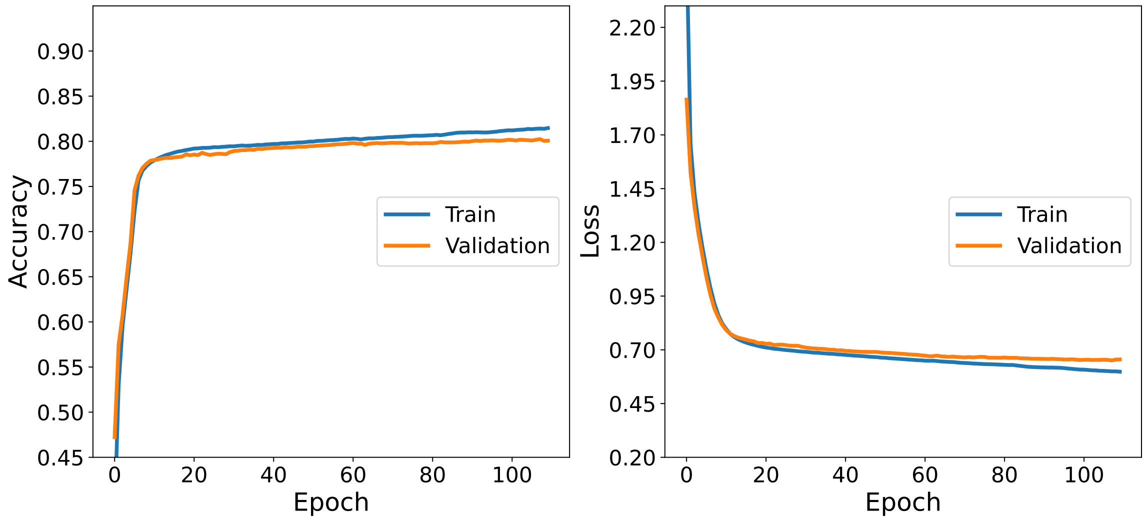

Figure S3.9: The training and validation history of the accuracy and loss for the MLP+RNN model using the set of ^{13}C spectral data with three specified experimental conditions with the neighbour approach.

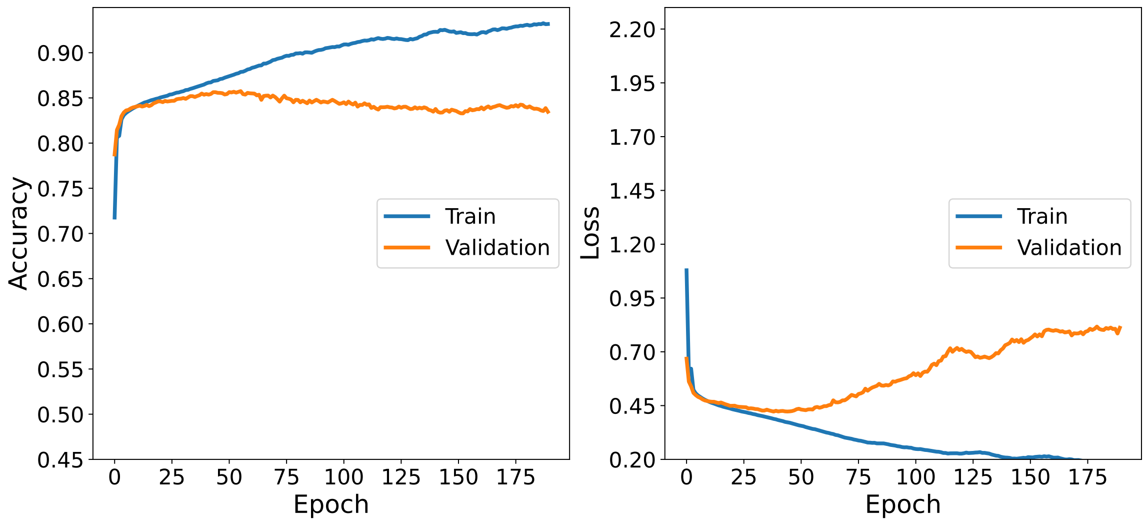

Figure S3.10: The training and validation history of the accuracy and loss for the MLP+LSTM model using the full set of ^{13}C spectral data with the functional group approach.

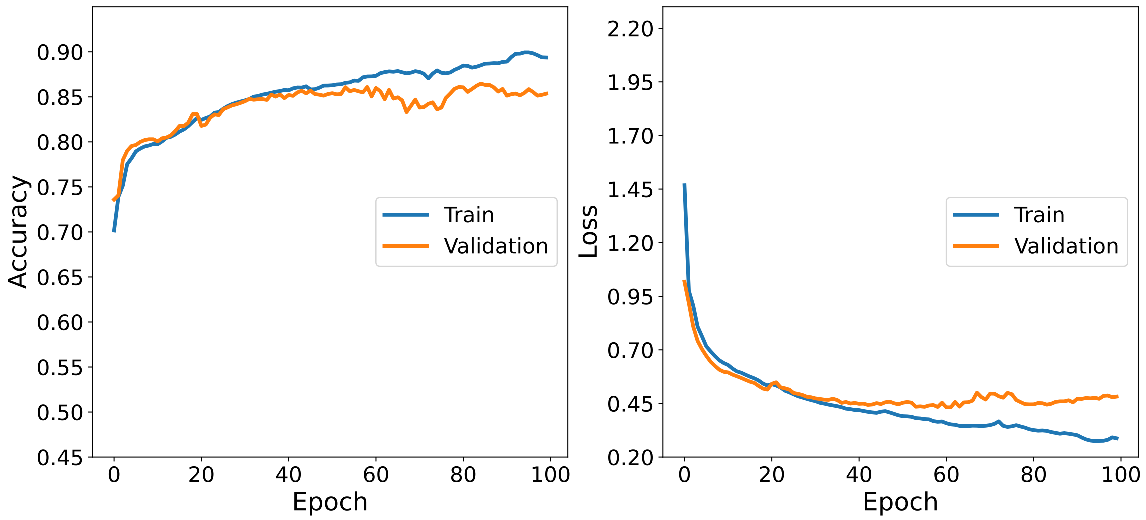

Figure S3.11: The training and validation history of the accuracy and loss for the MLP+LSTM model using the set of ^{13}C spectral data with three specified experimental conditions with the functional group approach.

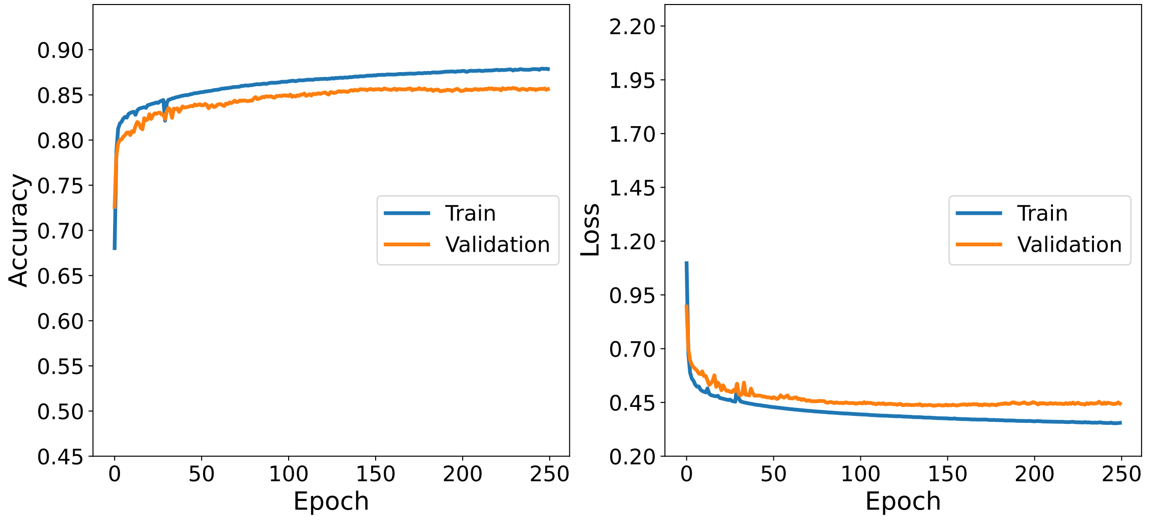

Figure S3.12: The training and validation history of the accuracy and loss for the CNN model using the full set of ^{13}C spectral data with the functional group approach.

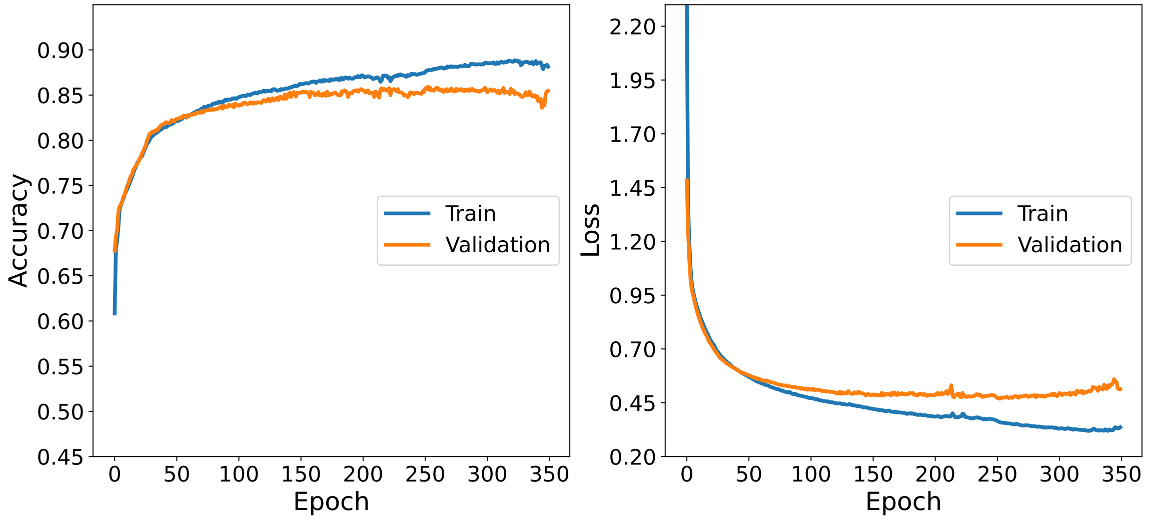

Figure S3.13: The training and validation history of the accuracy and loss for the CNN model using the set of ^{13}C spectral data with three specified experimental conditions with the functional group approach.

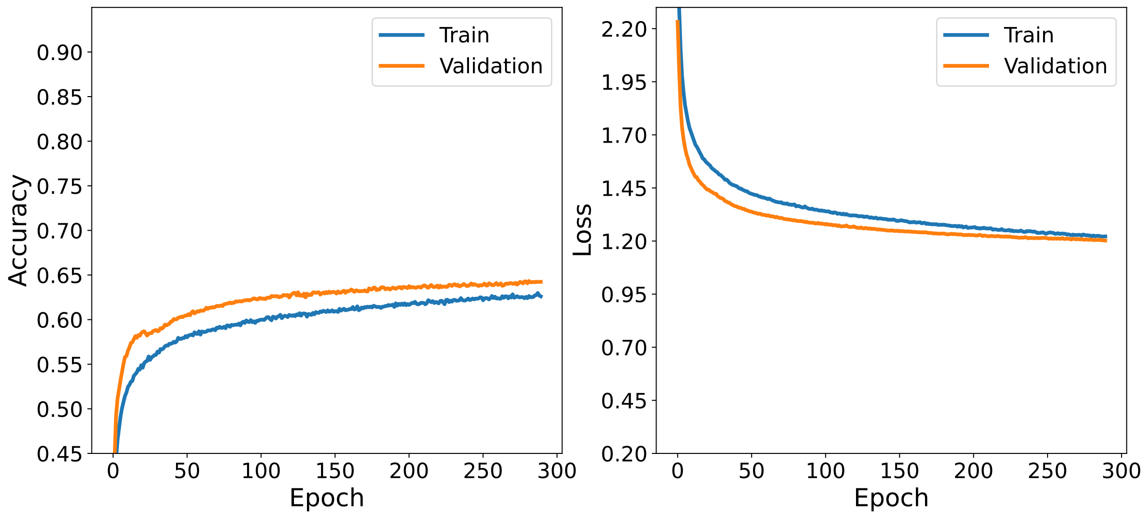

Figure S3.14: The training and validation history of the accuracy and loss for the MLP+LSTM model using the full set of ^{1}H spectral data with the neighbour approach.

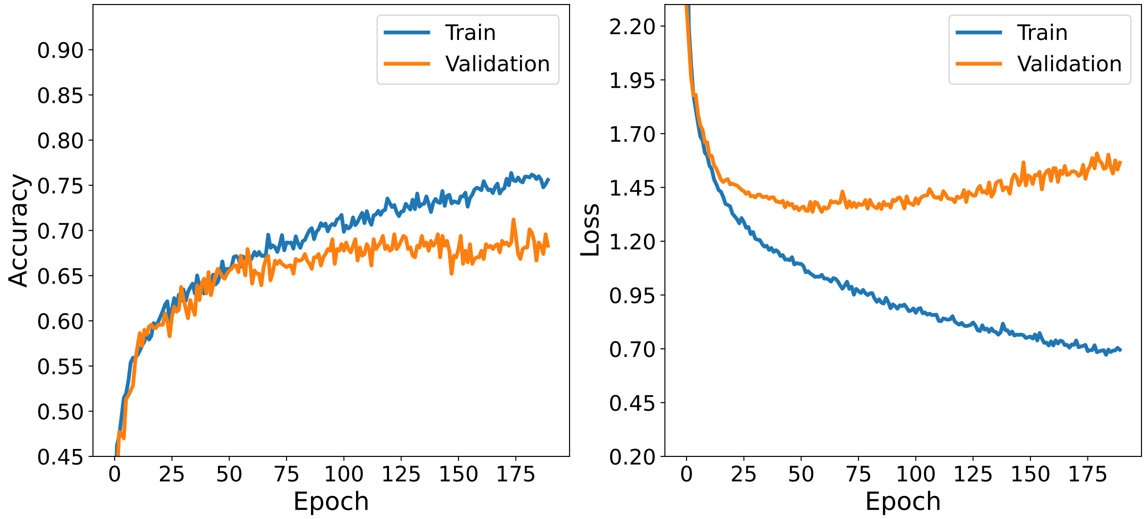

Figure S3.15: The training and validation history of the accuracy and loss for the MLP+LSTM model using the set of ^{1}H spectral data with three specified experimental conditions with the neighbour approach.

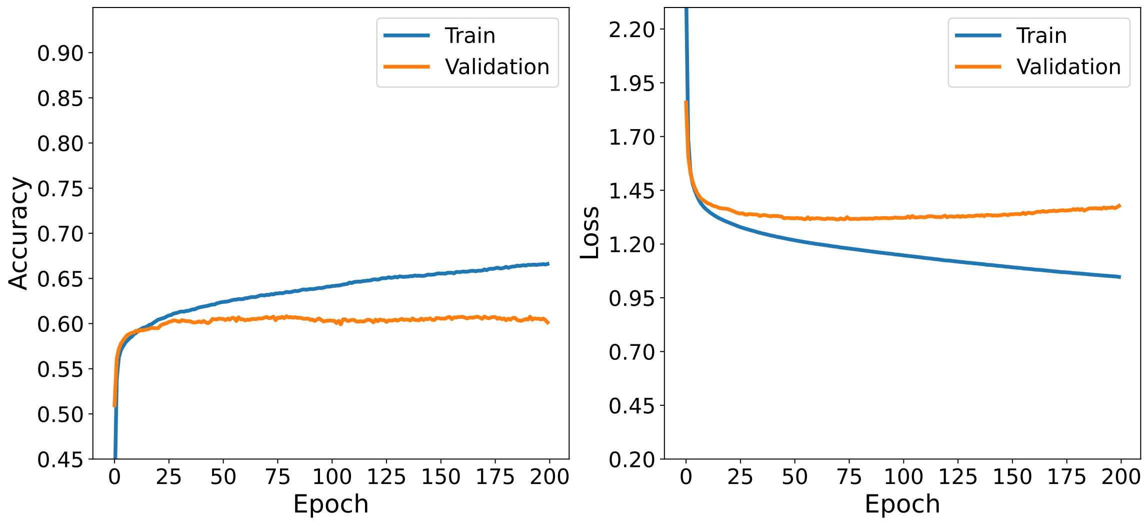

Figure S3.16: The training and validation history of the accuracy and loss for the CNN model using the full set of ^{1}H spectral data with the neighbour approach.

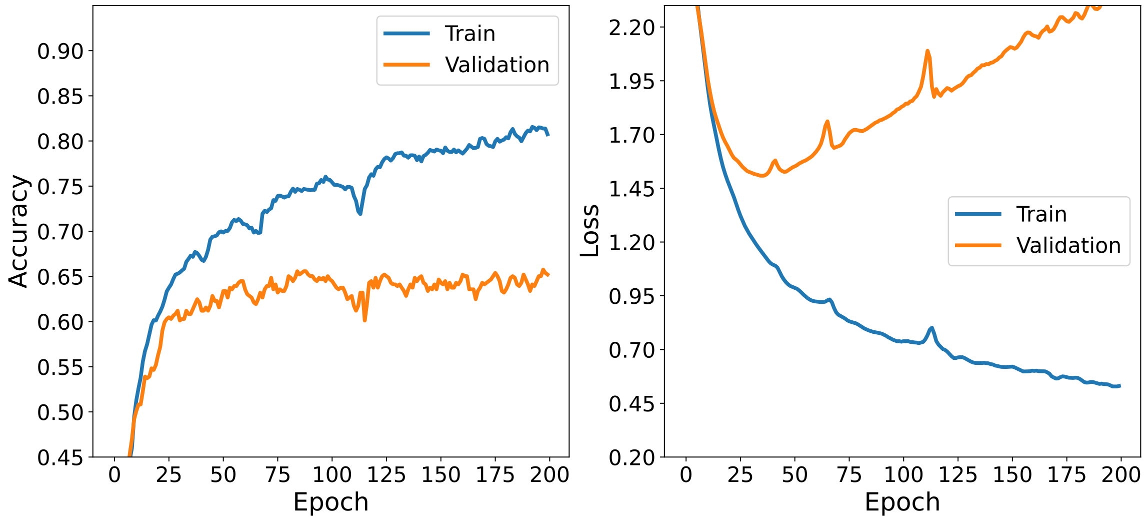

Figure S3.17: The training and validation history of the accuracy and loss for the CNN model using the set of ^{1}H spectral data with three specified experimental conditions with the neighbour approach.

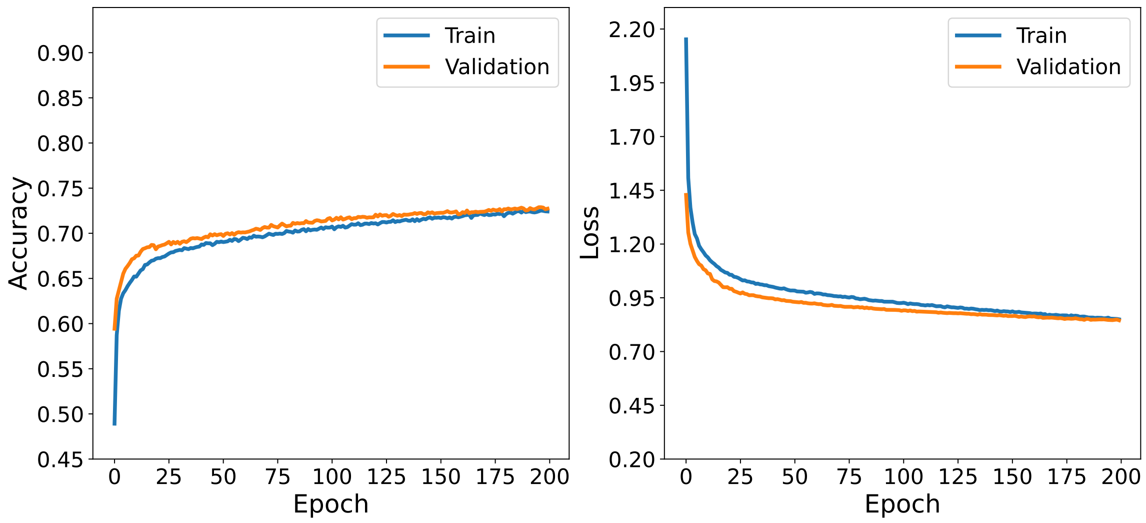

Figure S3.18: The training and validation history of the accuracy and loss for the MLP+LSTM model using the full set of ^{1}H spectral data with the functional group approach.

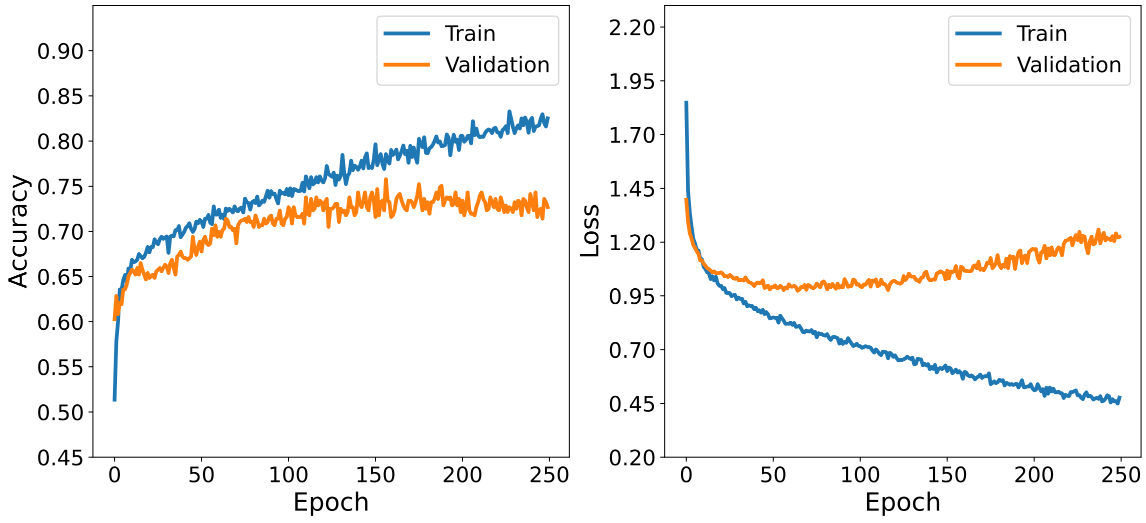

Figure S3.19: The training and validation history of the accuracy and loss for the MLP+LSTM model using the set of ^{1}H spectral data with three specified experimental conditions with the functional group approach.

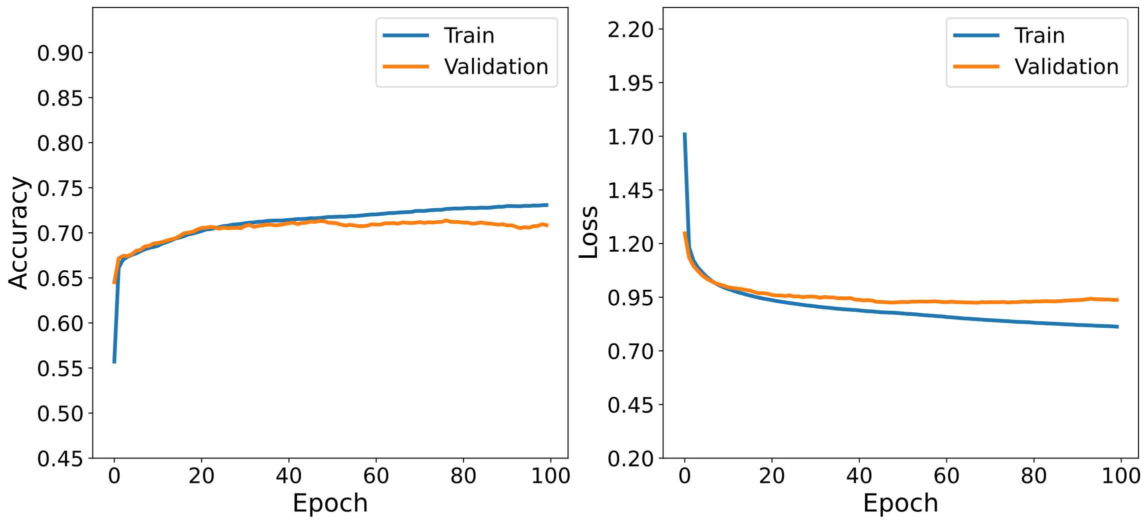

Figure S3.20: The training and validation history of the accuracy and loss for the CNN model using the full set of ^{1}H spectral data with the functional group approach.

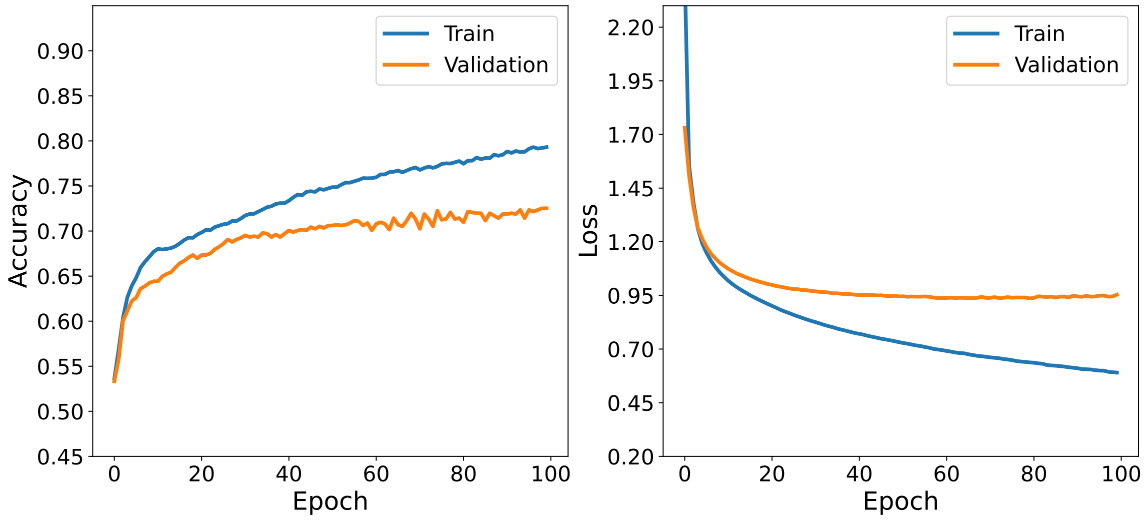

Figure S3.21: The training and validation history of the accuracy and loss for the CNN model using the set of ^{1}H spectral data with three specified experimental conditions with the functional group approach.

SI. 4 - Details of Case Studies that Demonstrate CNN Model Utility using the Neighbor Approach to Represent Spectrum-Structure Correlations

List of Case studies (compound names / modeling restrictions):

- S4.1 Beta-Sitosterol (using all ^{13}C NMR peak data provided by NMRshiftdb2)
- S4.2 Caffeine
- S4.3 Aspirin (Salicyclic Acid)
- S4.4 Cyclopropylbenzene (using all ^{13}C NMR peak data provided by NMRshiftdb2)

Full Details of Each Case Study

S4.1. Beta-Sitosterol (using all ^{13}C NMR peak data provided by NMRshiftdb2)

S4.1.1 Molecule (Ground Truth)

InChiKey: KZJWDPNRJALLNS-IDMGOKPUSA-N
Compound Name: Beta-Sitosterol
Chemical formula: $C_{29}H_{50}O$
Structural motif of molecule (screenshot from the NMRshiftdb2 database):

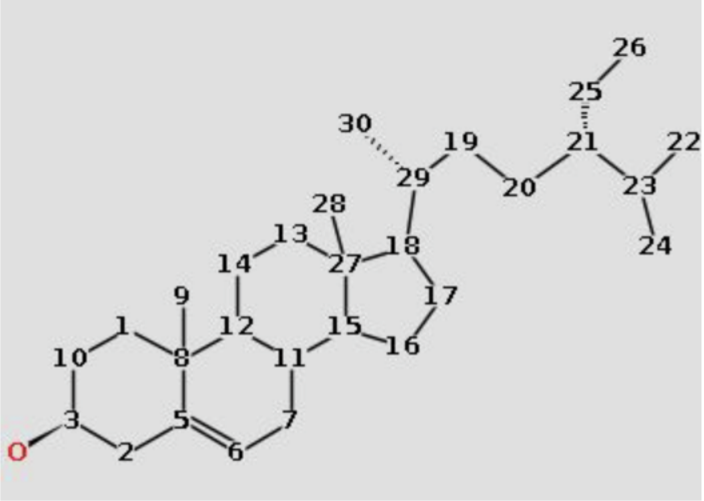

Figure S4.1.1: Chemical schematic of beta-Sitosterol

S4.1.2 Spectral Data used for Model Input from the NMRshiftdb2 Database Screenshots

Table S4.1.1 – Peak number, coupling constant, and chemical shift for ^{13}C NMR spectrum of beta-Sitosterol

Atom	Mult.(coupling const.)	Meas. Shift
1	T	37.30
2	T	42.30
3	D	71.70
5	S	140.80
6	D	121.60
7	T	31.96
8	S	36.50
9	Q	11.90
10	T	31.60
11	D	31.94
12	D	50.20
13	T	39.80
14	T	21.10
15	D	56.80
16	T	24.30
17	T	28.30
18	D	56.10
19	T	34.00
20	T	26.40
21	D	46.10
22	Q	19.10
23	D	29.00
24	Q	19.10
25	T	23.10
26	Q	12.30
27	T	42.30
28	Q	19.40
29	D	36.30
30	Q	18.80

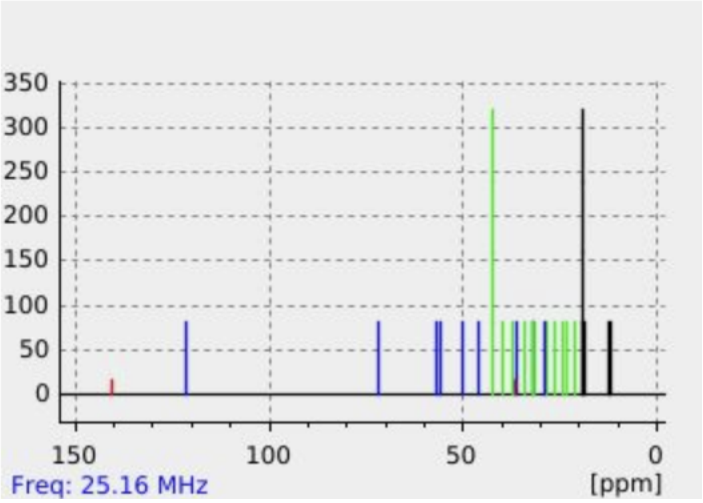

Figure S4.1.2: ^{13}C NMR spectrum of beta-Sitosterol reconstructed from the above peak data

S4.1.3 Reference Information for the Data about this Molecule

Nmrshiftdb2 molecule id: 2541

S4.1.4 The Full Input to the ML Model

Table S4.1.2 - Supporting experimental data used for ML model training

Field Strengths (MHz)	Temperatures (K)	Solvents
25.16	298	Chloroform-D1 (CDCl3)

For full input file, see associated Supporting Information document which carries the name: 'example41_actual_input'.

S4.1.5 Technical Details of the Model and Data

Model: CNN with optimized parameters (batch size: 100, No. of epochs: 120, optimizer: Adam, learning rate: 0.001, momentum (beta_1): 0.9)

Data: ^{13}C data with all three experimental conditions specified

S4.1.6 Results

Overall Model Accuracy: 86.2%

Table S4.1.3 - Predicted versus ground-truth (actual) chemical environment label (for neighbor approach) for each C atom when applying our CNN model

<table>
<tr><th>Actual
Labels</th><th>Actual
Visual</th><th>Predict
Visual</th><th>Predict
Labels</th></tr>
<tr><td>7 0 0 0 1 0 0 0</td><td colspan="2" rowspan="6">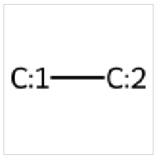
</td><td>7 0 0 0 1 0 0 0</td></tr>
<tr><td>7 0 0 0 1 0 0 0</td><td>7 0 0 0 1 0 0 0</td></tr>
<tr><td>7 0 0 0 1 0 0 0</td><td>7 0 0 0 1 0 0 0</td></tr>
<tr><td>7 0 0 0 1 0 0 0</td><td>7 0 0 0 1 0 0 0</td></tr>
<tr><td>7 0 0 0 1 0 0 0</td><td>7 0 0 0 1 0 0 0</td></tr>
<tr><td>7 0 0 0 1 0 0 0</td><td>7 0 0 0 1 0 0 0</td></tr>
<tr><td>7 7 0 0 1 1 0 0</td><td colspan="2" rowspan="5">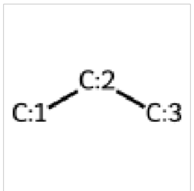
</td><td>7 7 0 0 1 1 0 0</td></tr>
<tr><td>7 7 0 0 1 1 0 0</td><td>7 7 0 0 1 1 0 0</td></tr>
<tr><td>7 7 0 0 1 1 0 0</td><td>7 7 0 0 1 1 0 0</td></tr>
<tr><td>7 7 0 0 1 1 0 0</td><td>7 7 0 0 1 1 0 0</td></tr>
<tr><td>7 7 0 0 1 1 0 0</td><td>7 7 0 0 1 1 0 0</td></tr>
<tr><td>7 7 7 0 1 1 1 0</td><td colspan="2">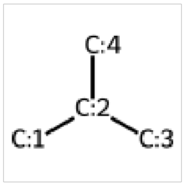
</td><td>7 7 7 0 1 1 1 0</td></tr>
<tr><td>7 7 0 0 1 1 0 0</td><td colspan="2">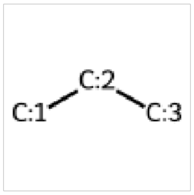
</td><td>7 7 0 0 1 1 0 0</td></tr>
<tr><td>7 7 7 0 1 1 1 0</td><td colspan="2">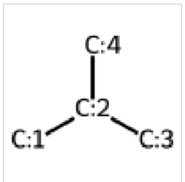
</td><td>7 7 7 0 1 1 1 0</td></tr>
<tr><td>7 7 0 0 1 1 0 0</td><td colspan="2" rowspan="2">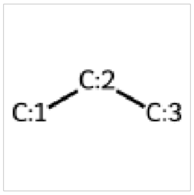
</td><td>7 7 0 0 1 1 0 0</td></tr>
<tr><td>7 7 0 0 1 1 0 0</td><td>7 7 0 0 1 1 0 0</td></tr>
<tr><td>7 7 7 0 1 1 1 0</td><td colspan="2">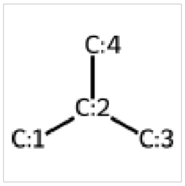
</td><td>7 7 7 0 1 1 1 0</td></tr>
<tr><td>7 7 7 7 1 1 1 1</td><td colspan="2">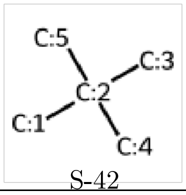
</td><td>7 7 7 7 1 1 1 1</td></tr>
</table>

<table>
<tr><td>7 7 0 0 1 1 0 0</td><td rowspan="2">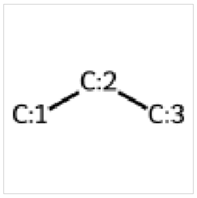
</td><td rowspan="4">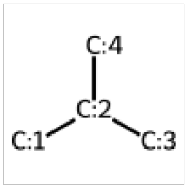
</td><td>7 7 7 0 1 1 1 0</td></tr>
<tr><td>7 7 0 0 1 1 0 0</td><td>7 7 7 0 1 1 1 0</td></tr>
<tr><td>7 7 0 0 1 1 0 0</td><td>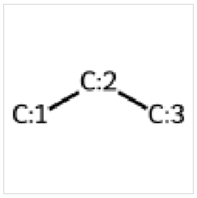
</td><td>7 7 7 0 1 1 1 0</td></tr>
<tr><td>7 7 7 7 1 1 1 1</td><td>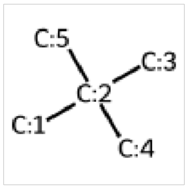
</td><td>7 7 7 0 1 1 1 0</td></tr>
<tr><td>7 7 7 0 1 1 1 0</td><td colspan="2" rowspan="4">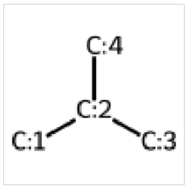
</td><td>7 7 7 0 1 1 1 0</td></tr>
<tr><td>7 7 7 0 1 1 1 0</td><td>7 7 7 0 1 1 1 0</td></tr>
<tr><td>7 7 7 0 1 1 1 0</td><td>7 7 7 0 1 1 1 0</td></tr>
<tr><td>7 7 7 0 1 1 1 0</td><td>7 7 7 0 1 1 1 0</td></tr>
<tr><td>7 7 19 0 1 1 1 0</td><td colspan="2">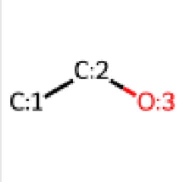
</td><td>7 7 19 0 1 1 1 0</td></tr>
<tr><td>7 7 0 0 1 2 0 0</td><td colspan="2">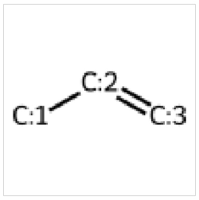
</td><td>7 7 0 0 1 2 0 0</td></tr>
<tr><td>7 7 7 0 1 1 2 0</td><td colspan="2">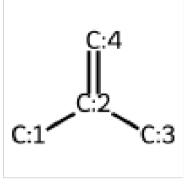
</td><td>7 7 7 0 1 1 2 0</td></tr>
</table>

S4.1.7 Supporting comments / observations about the results

If the atom number is not specified in the chemical schematics within the table, label '2' always refers to the atom number that corresponds to the principal atom whose neighbors are predicted.

For the textual digits, a label of 7 represents a carbon atom and a label of 19 represents an oxygen atom. The labels are recovered from the raw output of the model, as the raw output is one-hot encoded. The visualizations are generated using rdkit.Chem and rdkit.Draw in python. If there is a zero in the first 4 digits, it means that the target carbon atom is also bonded with a hydrogen.

S4.2 Caffeine

S4.2.1 Molecule (Ground Truth)

InChIKey: RYYVLZVUVIJVGH-UHFFFAOYSA-N

Name: 1,3,7-Trimethylpurine-2,6-dione; 1,3,7-Trimethylxanthine; caffeine; Coffein; Gua

Chemical formula: $C_8H_{10}N_4O_2$

Choice of NMR Solvent and Field Strength: Dimethylsulphoxide-D6 solvent, field strength of 100.5679

Structural motif of molecule (screenshot from the NMRshiftdb2 database (see below, left))

S4.2.2 Spectral Data used for Model Input from the NMRshiftdb2 Database Screenshots

Type: 13C 100.5679702758789

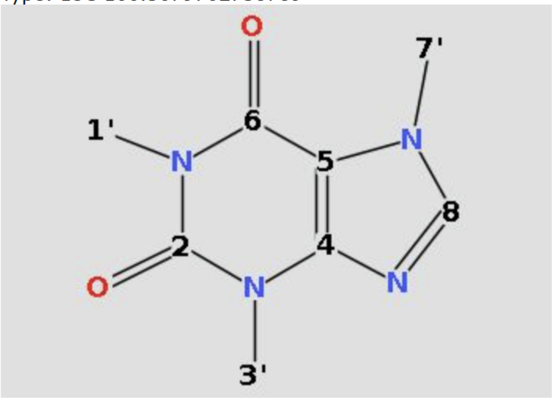

Atom	Mult. (coupling const.)	Meas. Shift
1'	Q	27.87
2	S	151.58
3'	Q	30.14
4	S	148.65
5	S	107.13
6	S	155.08
7'	Q	33.59
8	D	143.26

Show spectrum in full size

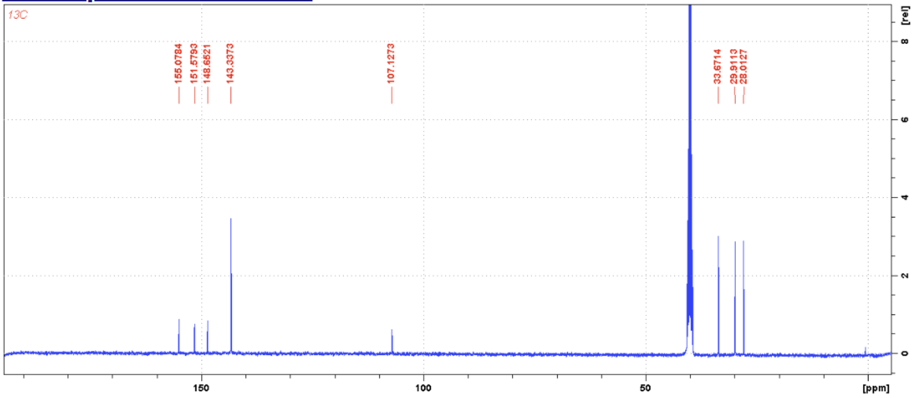

Figure S4.2.1: (top left) Chemical schematic of the caffeine molecule; (top right) Peak number, coupling constant, and chemical shift for ^{13}C NMR spectrum of caffeine; (bottom) ^{13}C NMR spectrum of caffeine reconstructed from the above peak data.

S4.2.3 Reference Information for the Data about this Molecule

Nmrshiftdb2 molecule id is 10016316

S4.2.4 The Full Input to the ML Model

Table S4.2.1 - Supporting experimental data used for ML model training.

Field Strengths (MHz)	Temperatures (K)	Solvents
100.568	298	Dimethylsulphoxide-D6

Table S4.2.2 - For full input file for ML model application.

Field Strengths	Temperatures	Solvents	Shifts _x	Coupling _x	Other Shifts	Other Coupling
0.143669	0.922601	10	30.14	4	27.87, 30.14, 33.59, 107.13, 143.26, 148.65, 151.58, 155.08	4, 4, 4, 1, 2, 1, 1, 1
0.143669	0.922601	10	33.59	4	27.87, 30.14, 33.59, 107.13, 143.26, 148.65, 151.58, 155.08	4, 4, 4, 1, 2, 1, 1, 1
0.143669	0.922601	10	107.13	1	27.87, 30.14, 33.59, 107.13, 143.26, 148.65, 151.58, 155.08	4, 4, 4, 1, 2, 1, 1, 1
0.143669	0.922601	10	143.26	2	27.87, 30.14, 33.59, 107.13, 143.26, 148.65, 151.58, 155.08	4, 4, 4, 1, 2, 1, 1, 1
0.143669	0.922601	10	148.65	1	27.87, 30.14, 33.59, 107.13, 143.26, 148.65, 151.58, 155.08	4, 4, 4, 1, 2, 1, 1, 1
0.143669	0.922601	10	151.58	1	27.87, 30.14, 33.59, 107.13, 143.26, 148.65, 151.58, 155.08	4, 4, 4, 1, 2, 1, 1, 1
0.143669	0.922601	10	155.08	1	27.87, 30.14, 33.59, 107.13, 143.26, 148.65, 151.58, 155.08	4, 4, 4, 1, 2, 1, 1, 1

S4.2.5 Technical Details of the Model and Data

Model: CNN with optimized parameters (batch size: 100, No. of epochs: 120, optimizer: Adam, learning rate: 0.001, momentum (beta_1): 0.9)

Data: ^{13}C data with all three experimental conditions specified

S4.2.6 Results

Overall Model Accuracy: 72% (6/8 atoms matched)

Table S4.2.3 - Predicted versus ground-truth (actual) chemical environment label (for neighbor approach) for each C atom when applying our CNN model.

Actual Labels	Actual visual	Predict Visual	Predict Labels
17 0 0 0 1 0 0 0	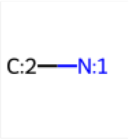		17 0 0 0 1 0 0 0
17 0 0 0 1 0 0 0			17 0 0 0 1 0 0 0
17 0 0 0 1 0 0 0			17 0 0 0 1 0 0 0
7 7 17 0 1 2 1 0	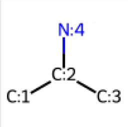	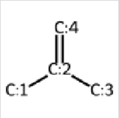	7 7 7 0 1 1 2 0
17 17 0 0 1 2 0 0	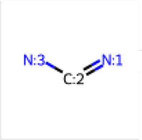		17 17 0 0 1 2 0 0
7 17 17 0 2 1 1 0	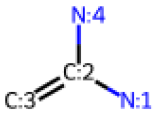		7 17 17 0 2 1 1 0
17 17 19 0 1 1 2 0	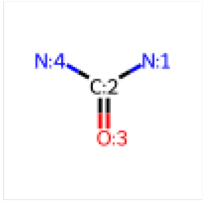	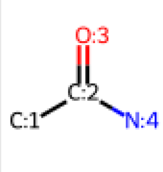	7 17 19 0 1 1 2 0
7 17 19 0 1 1 2 0	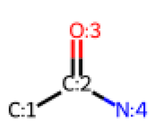		7 17 19 0 1 1 2 0

S4.2.7 Supporting Comments / Observations about the Results

17 is N, 19 is O, 7 is C where present within the first four digits of the chemical molecule labels (using the neighbor approach).

S4.3 Aspirin (Salicyclic Acid)

S4.3.1 Molecule (Ground Truth)

InChI Key: BSYNRYMUTXBXSQ-UHFFFAOYSA-N

Chemical name: 2-acetoxybenzoic acid; acenterine; acetosal; acetylsalicylic acid; acylpyrin; aspirin; colfarit; enterosarein; o-acetoxybenzoic acid

Chemical formula: $C_9H_8O_4$

Structural motif of molecule (screenshot from the NMRshiftdb2 database (see below, left))

S4.3.2 Spectral Data used for Model Input from the NMRshiftdb2 Database Screenshots

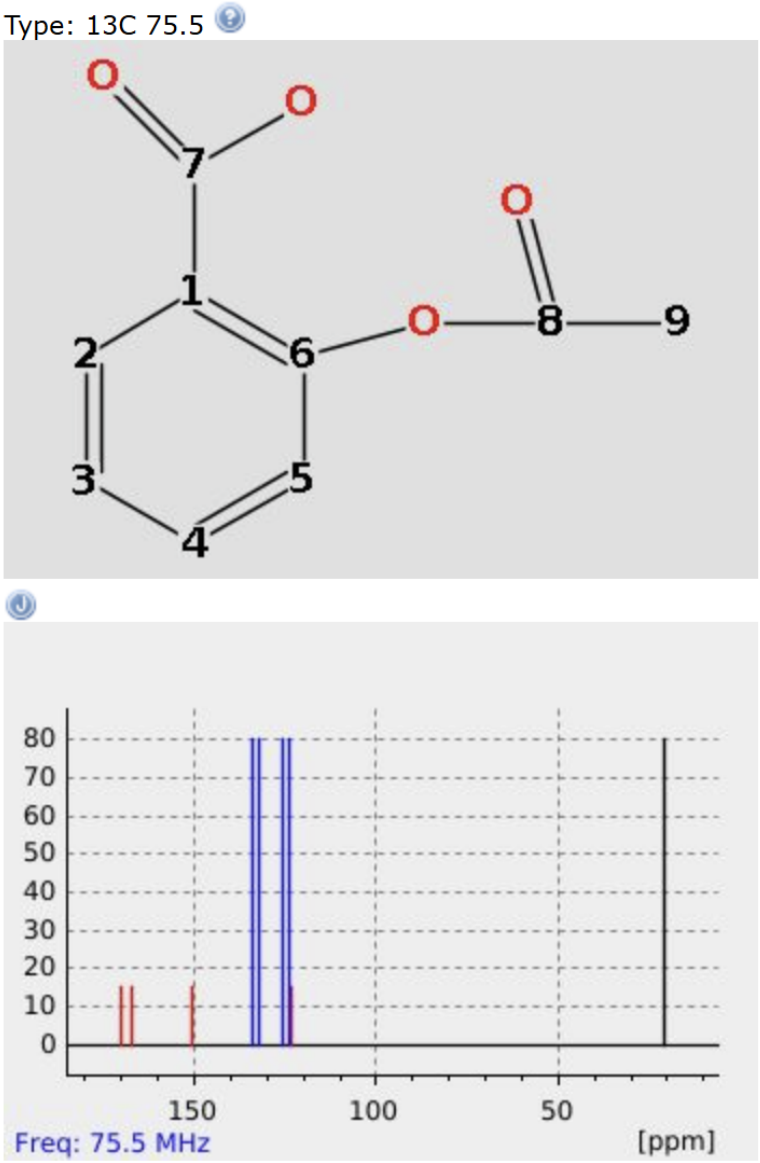

Atom	Mult. (coupling const.)	Meas. Shift
1	S	123.4
2	D	132.2
3	D	126.0
4	D	134.0
5	D	123.7
6	S	150.9
7	S	167.3
8	S	170.0
9	Q	21.1

Figure S4.4.1: (top left) Chemical schematic of the aspirin molecule; (top right) Peak number, coupling constant, and chemical shift for ^{13}C NMR spectrum of aspirin; (bottom) ^{13}C NMR spectrum of aspirin reconstructed from the above peak data.

S4.3.3 Reference Information for the Data about this Molecule

Nmrshiftdb2 molecule id is 20038075

S4.3.4 The Full Input to the ML Model

Table S4.3.1 - Supporting experimental data used for ML model training.

Field Strengths (MHz)	Temperatures (K)	Solvents
500	298	Chloroform-D1 (CDCl3)

Table S4.3.2 - For full input file for ML model application of aspirin.

Field Strengths	Temperatures	Solvents	Shifts _x	Coupling _x	Other Shifts	Other Coupling
0.714286	0.922601	4	21.1	4	21.1, 123.4, 123.7, 126.0, 132.2, 134.0, 150.9, 167.3, 170.0	4, 1, 2, 2, 2, 2, 1, 1, 1
0.714286	0.922601	4	123.4	1	21.1, 123.4, 123.7, 126.0, 132.2, 134.0, 150.9, 167.3, 170.0	4, 1, 2, 2, 2, 2, 1, 1, 1
0.714286	0.922601	4	123.7	2	21.1, 123.4, 123.7, 126.0, 132.2, 134.0, 150.9, 167.3, 170.0	4, 1, 2, 2, 2, 2, 1, 1, 1
0.714286	0.922601	4	126	2	21.1, 123.4, 123.7, 126.0, 132.2, 134.0, 150.9, 167.3, 170.0	4, 1, 2, 2, 2, 2, 1, 1, 1
0.714286	0.922601	4	132.2	2	21.1, 123.4, 123.7, 126.0, 132.2, 134.0, 150.9, 167.3, 170.0	4, 1, 2, 2, 2, 2, 1, 1, 1
0.714286	0.922601	4	134	2	21.1, 123.4, 123.7, 126.0, 132.2, 134.0, 150.9, 167.3, 170.0	4, 1, 2, 2, 2, 2, 1, 1, 1
0.714286	0.922601	4	150.9	1	21.1, 123.4, 123.7, 126.0, 132.2, 134.0, 150.9, 167.3, 170.0	4, 1, 2, 2, 2, 2, 1, 1, 1
0.714286	0.922601	4	167.3	1	21.1, 123.4, 123.7, 126.0, 132.2, 134.0, 150.9, 167.3, 170.0	4, 1, 2, 2, 2, 2, 1, 1, 1
0.714286	0.922601	4	170	1	21.1, 123.4, 123.7, 126.0, 132.2, 134.0, 150.9, 167.3, 170.0	4, 1, 2, 2, 2, 2, 1, 1, 1

S4.3.5 Technical Details of the Model and Data

Model: CNN with optimized parameters (batch size: 100, No. of epochs: 120, optimizer: Adam, learning rate: 0.001, momentum (beta_1): 0.9)

Data: ^{13}C data with all three experimental conditions specified

S4.3.6 Results

Overall Model Accuracy: 100% (chemical environments matched for all chemically non-equivalent atoms)

Table S4.3.3 - Predicted versus ground-truth (actual) chemical environment label (for neighbor approach) for each C atom when applying our CNN model.

<table>
<tr><th>Actual
Labels</th><th>Actual
visual</th><th>Predict
Visual</th><th>Predict
Labels</th></tr>
<tr><td>7 0 0 0 1 0 0 0</td><td colspan="2">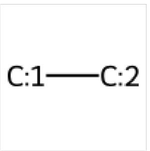</td><td>7 0 0 0 1 0 0 0</td></tr>
<tr><td>7 7 7 0 1 1 2 0</td><td colspan="2">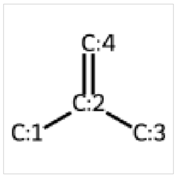</td><td>7 7 7 0 1 1 2 0</td></tr>
<tr><td>7 7 0 0 1 2 0 0</td><td colspan="2" rowspan="4">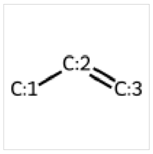</td><td>7 7 0 0 1 2 0 0</td></tr>
<tr><td>7 7 0 0 1 2 0 0</td><td>7 7 0 0 1 2 0 0</td></tr>
<tr><td>7 7 0 0 1 2 0 0</td><td>7 7 0 0 1 2 0 0</td></tr>
<tr><td>7 7 0 0 1 2 0 0</td><td>7 7 0 0 1 2 0 0</td></tr>
<tr><td>6 7 7 0 1 1 1 0</td><td colspan="2" rowspan="2">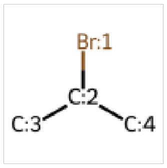</td><td>6 7 7 0 1 1 1 0</td></tr>
<tr><td>6 7 7 0 1 1 1 0</td><td>6 7 7 0 1 1 1 0</td></tr>
<tr><td>7 19 19 0 1 1 2 0</td><td colspan="2" rowspan="2">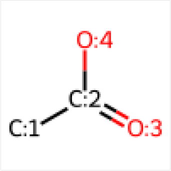</td><td>7 19 19 0 1 1 2 0</td></tr>
<tr><td>7 19 19 0 1 1 2 0</td><td>7 19 19 0 1 1 2 0</td></tr>
</table>

S4.4 Cyclopropylbenzene (using all ^{13}C NMR peak data provided by NMR-shiftdb2)

S4.4.1 Molecule (Ground Truth)

InChI Key: VFSFCYAQBIPUSL-UHFFFAOYSA-N

Chemical name: cyclopropylbenzene

Chemical formula: C_9H_{10}

Structural motif of molecule (screenshot from the NMRshiftdb2 database (see below, left))

S4.4.2 Spectral Data used for Model Input from the NMRshiftdb2 Database Screenshots

Type: 13C Unreported

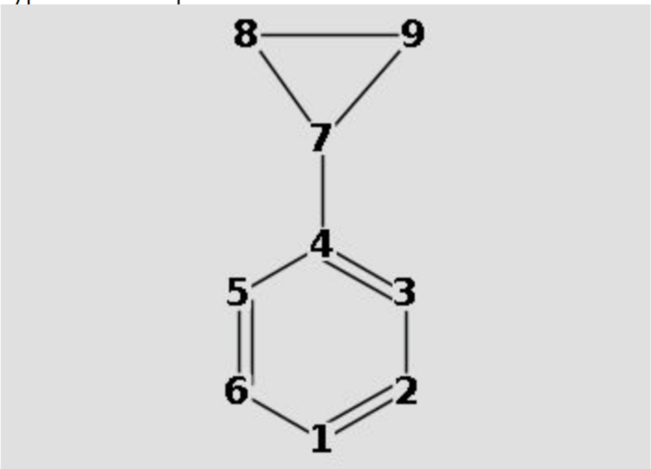

Atom	Mult.(coupling const.)	Meas. Shift
1	D	125.2
2	D	128.1
3	D	125.5
4	S	143.7
5	D	125.5
6	D	128.1
7	D	15.4
8	T	9.1
9	T	9.1

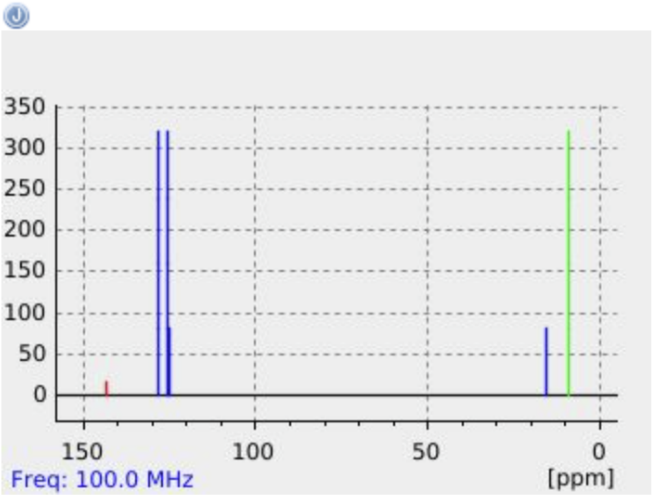

Figure S4.4.1: (top left) Chemical schematic of the cyclopropylbenzene molecule; (top right) Peak number, coupling constant, and chemical shift for ^{13}C NMR spectrum of cyclopropylbenzene; (bottom) ^{13}C NMR spectrum of cyclopropylbenzene reconstructed from the above peak data.

S4.4.3 Reference Information for the Data about this Molecule

Nmrshiftdb2 molecule id is 10016581

S4.4.4 The Full Input to the ML Model

There were no supporting experimental data (solvent, temperature, field strength) used for training.

Table S4.4.1 - For full input file for ML model application of cyclopropylbenzene.

Field Strengths	Temperatures	Solvents	Shifts_x	Coupling_x	Other Shifts	Other Coupling
0	0	0	9.1	3	9.1, 9.1, 15.4, 125.2, 125.5, 125.5, 128.1, 128.1, 143.7	3, 3, 2, 2, 2, 2, 2, 2, 1
0	0	0	9.1	3	9.1, 9.1, 15.4, 125.2, 125.5, 125.5, 128.1, 128.1, 143.7	3, 3, 2, 2, 2, 2, 2, 2, 1
0	0	0	15.4	2	9.1, 9.1, 15.4, 125.2, 125.5, 125.5, 128.1, 128.1, 143.7	3, 3, 2, 2, 2, 2, 2, 2, 1
0	0	0	125.2	2	9.1, 9.1, 15.4, 125.2, 125.5, 125.5, 128.1, 128.1, 143.7	3, 3, 2, 2, 2, 2, 2, 2, 1
0	0	0	125.5	2	9.1, 9.1, 15.4, 125.2, 125.5, 125.5, 128.1, 128.1, 143.7	3, 3, 2, 2, 2, 2, 2, 2, 1
0	0	0	125.5	2	9.1, 9.1, 15.4, 125.2, 125.5, 125.5, 128.1, 128.1, 143.7	3, 3, 2, 2, 2, 2, 2, 2, 1
0	0	0	128.1	2	9.1, 9.1, 15.4, 125.2, 125.5, 125.5, 128.1, 128.1, 143.7	3, 3, 2, 2, 2, 2, 2, 2, 1
0	0	0	128.1	2	9.1, 9.1, 15.4, 125.2, 125.5, 125.5, 128.1, 128.1, 143.7	3, 3, 2, 2, 2, 2, 2, 2, 1
0	0	0	143.7	1	9.1, 9.1, 15.4, 125.2, 125.5, 125.5, 128.1, 128.1, 143.7	3, 3, 2, 2, 2, 2, 2, 2, 1

S4.4.5 Technical Details of the Model and Data

Model: CNN with optimized parameters (batch size: 500, No. of epochs: 150, optimizer: Adamax, learning rate: 0.001, momentum (beta_1): 0.9)

Data: Complete ^{13}C data (with or without any experimental conditions specified)

S4.4.6 Results

Overall Model Accuracy: 100% (chemical environments matched for all chemically non-equivalent atoms)

Table S4.4.2 - Predicted versus ground-truth (actual) chemical environment label (for neighbor approach) for each C atom when applying our CNN model.

Actual Labels	Actual visual	Predict Visual	Predict Labels
7 7 0 0 1 1 0 0	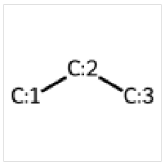		7 7 0 0 1 1 0 0
7 7 0 0 1 1 0 0			7 7 0 0 1 1 0 0
7 7 7 0 1 1 1 0	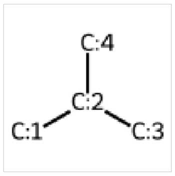		7 7 7 0 1 1 1 0
7 7 0 0 1 2 0 0	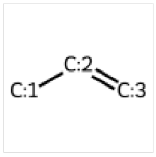		7 7 0 0 1 2 0 0
7 7 0 0 1 2 0 0			7 7 0 0 1 2 0 0
7 7 0 0 1 2 0 0			7 7 0 0 1 2 0 0
7 7 0 0 1 2 0 0			7 7 0 0 1 2 0 0
7 7 0 0 1 2 0 0			7 7 0 0 1 2 0 0
7 7 7 0 1 1 2 0	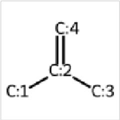		7 7 7 0 1 1 2 0

S4.4.7 Supporting Comments / Observations about the Results

Any elements that are not N O C are labeled as 32 in the chemical schematics within the table above. Regarding the text digits, 7 is still carbon, 19 is still O and 17 is Nitrogen within the chemical element labelling scheme of the neighbor approach.
